# Multicellular microtissues from fused ligament- and bone-cell spheroids relevant to enthesis repair

**DOI:** 10.1016/j.mtbio.2026.103129

**Published:** 2026-04-15

**Authors:** Francesca Giacomini, Shivesh Anand, Steven Vermeulen, David Barata, Zeinab Niloofar Tahmasebi Birgani, Pieter J. Emans, Carmen López-Iglesias, Lorenzo Moroni, Carlos Mota, Stefan Giselbrecht, Pamela Habibović, Roman Truckenmüller

**Affiliations:** a MERLN Institute for Technology-Inspired Regenerative Medicine, Maastricht University, Universiteitssingel 40, Maastricht, 6229 ER, the Netherlands; b Department of Orthopedic Surgery, Joint-Preserving Clinic, Care and Public Health Research Institute (CAPHRI), Maastricht University Medical Center (MUMC)+, P. Debyelaan 25, Maastricht, 6229 HX, the Netherlands; c Microscopy CORE Lab, Maastricht MultiModal Molecular Imaging Institute (M4I), Maastricht University, Universiteitssingel 50, Maastricht, 6229 ER, the Netherlands

**Keywords:** Enthesis, Microtissues, Spheroid fusion, *de novo* tissue formation, High-throughput screening, Acoustic/ultrasound stimulation

## Abstract

The enthesis, the point where a tendon or ligament attaches to bone, is a graded fibrocartilaginous interface that poorly regenerates after injury. Here, we present a modular and scaffold-free strategy for engineering microtissues relevant to enthesis repair by fusing anterior cruciate ligament-derived spheroids with spheroids from osteogenically differentiated human mesenchymal stromal cells. We show that the maturation state of the constituent spheroids governs fusion dynamics and spatial organization, enabling the controlled formation of ligament-, fibrocartilage-, and bone-like regions within a single, radially/concentrically organized construct. Within 10 days, the fused tissues display locally distributed lineage-specific markers and type X collagen localized at the interface between the osteogenically-derived core and ligamentous shell. The latter is indicative of the *de novo* formation of a fibrocartilage-like region between these regions. The system is scalable by simply adjusting the spheroid number. It supports external mechanical stimulation via ultrasound. The acoustic cues further promote extracellular matrix deposition and tissue growth while maintaining structural integrity. This readily implementable heterotypic spheroid platform offers an *in vitro* model for studying enthesis mechanobiology, screening therapeutic compounds, and evaluating microscale biomaterials, with translational potential as injectable or bioprintable building blocks for enthesis repair.

## Graphical abstract

**Figure:**
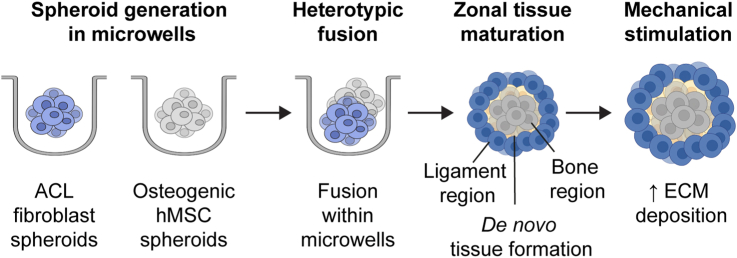

## 1 Introduction

The enthesis is the anatomical junction where a tendon or ligament integrates with bone, forming a structurally continuous interface spanning non-calcified tendon or ligament, unmineralized and mineralized fibrocartilage, and subchondral bone [[1], [2], [3], [4]]. This zonal organization is reflected at the cellular level. Tenocytes or ligamentocytes are predominant in the soft domain and synthesize type I collagen (COL-I). Fibrochondrocytes in the transitional zones produce type II collagen (COL-II), aggrecan, and, upon hypertrophy, type X collagen (COL-X) associated with mineralization. Finally, osteoblasts and osteocytes populate the osseous region and regulate matrix deposition and remodeling [1,5]. Functionally, this graded structure anchors soft tissues to the skeleton, transmits muscle forces, and contributes to joint stability [6].

The enthesis is frequently injured across all age groups: in young, active individuals through sports trauma, such as anterior cruciate ligament (ACL) injuries [7], and in older adults, through degenerative processes [8]. Following injury, the natural healing process is often inadequate, resulting in the inability to fully restore the structure and function of the tissue [5,[9], [10], [11]]. Similarly, surgical reattachment rarely regenerates the native fibrocartilaginous transition; instead, fibrotic scar tissue with inferior mechanical properties commonly forms, predisposing to re-tear [10,11]. These limitations highlight the need for strategies capable of restoring the graded structure of the native enthesis. Addressing this challenge requires insights from both fundamental and translational research. Fundamental studies using multiscale imaging, spectroscopy, and mechanical testing have mapped zonal ultrastructure and quantified how tendon fiber geometry and composition govern stress transfer [[12], [13], [14]]. Developmental and single-cell analyses are revealing lineage trajectories and cell-state plasticity [15]. Translational efforts focus on improving repair, for example, through graded, multicompartmental and multiphasic biomaterial scaffolds [16], and spatially programmed bioactive cues to guide zonal maturation [17]. Animal models remain essential for investigating tissue regeneration, but are expensive, often limited in their translatability to human physiology, and raise ethical concerns regarding animal welfare. In line with the 3Rs principles (Replacement, Reduction, and Refinement), these limitations have accelerated the demand for human-relevant *in vitro* systems that can recapitulate tissue physiology and pathology, including organoid-based biology and disease modeling [18].

*In vitro* enthesis models have progressed from two-dimensional (2D) co-cultures [19] to more advanced systems based on engineered gradients [20], biphasic and triphasic hydrogels [21], fiber-reinforced constructs [22], and microphysiological “enthesis-on-chip” models [23]. These systems enable spatial control of structure and composition of the engineered extracellular matrix (ECM) and allow for the application of defined mechanical and fluidic boundary conditions. However, despite these advances, many current models are technically demanding, limiting their scalability and practical accessibility. In contrast, spheroid-based approaches, which are well established in other tissue contexts for reproducible and parallelized experimentation as well as compatibility with bioimaging and downstream analyses, remain underexplored in enthesis biology.

**Fig. 1:**
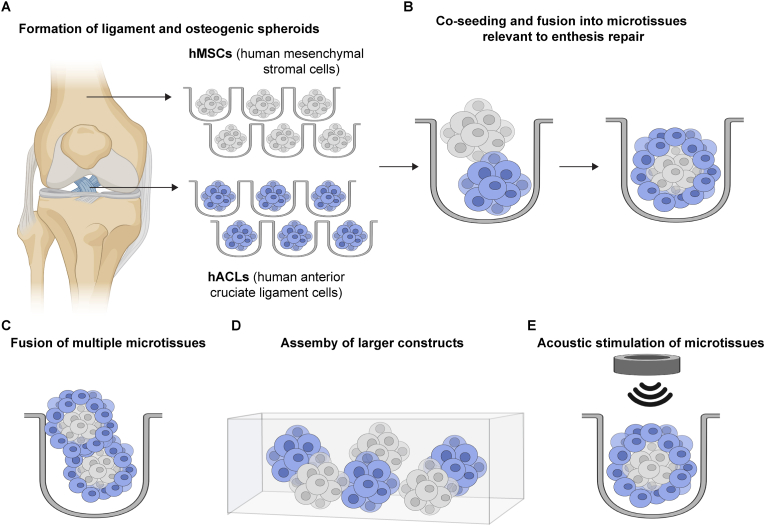
**Engineered microtissues relevant to enthesis repair generated by spheroid fusion.** A) Patient-derived anterior cruciate ligament (ACL) cells (fibroblasts) and human mesenchymal stromal cells (hMSCs) were separately aggregated in non-adherent microwell arrays to form ligament-like and osteogenic spheroids, respectively. **B)** ACL-derived spheroids and osteogenically differentiated hMSC spheroids were co-seeded within microwells, where they self-assembled into multicellular aggregates. After 10 days of co-culture, the fused spheroids developed a core-shell architecture and expressed early enthesis markers. **C** and **D)** Progressive fusion of microtissues occurred at both (C) microscale and (D) milliscale, generating continuous, spatially organized ligament-bone interface constructs. **E)** Ligament-bone interface microtissues were subjected to acoustic stimulation to evaluate mechanoresponsiveness and extracellular matrix deposition.

In this study, we present a modular spheroid-fusion strategy to create ligament-bone interface microtissues, providing an *in vitro* platform for studying cues relevant to enthesis repair (Fig. 1). These ligament-bone interface microtissues are created by fusing ligamentous and osteogenically derived spheroids. The maturation state of the constituent spheroids governs fusion kinetics and spatial organization, enabling control over interface formation. Within 10 days, the resulting constructs express ligament and bone lineage markers, with localized COL-X deposition at the junction indicating the *de novo* formation of a fibrocartilage-like region. The system is scalable, as microtissue size can be increased by simply varying the spheroid number. To exploit the mechanoresponsive nature of the enthesis, we apply acoustic stimulation, which enhances ECM deposition and tissue growth without compromising structural integrity. Together, our results demonstrate a reproducible, scaffold-free *in vitro* model of the ligament-bone interface. While these data do not yet fully recapitulate the structural and compositional gradients of the enthesis, the model provides a foundation for developing a versatile platform for mechanobiology studies, screening of interface-targeted therapeutics, and microscale biomaterials evaluation.

## 2 Materials and methods

### 2.1 Preparatory cell culture

Human ACL fibroblasts/cells were obtained from a 62-year-old female patient who underwent full replacement after traumatic injury and had given consent from Maastricht University Medical Center (Medisch Ethische Toetsingscommissie; METC number 2017-0183). Cell isolation was performed as previously described [24]. The cells were seeded in T-flasks at a density of 5000 cells cm^−2^ and expanded in basic cell culture medium for ligament cells (BM_L_) composed of Dulbecco's Modified Eagle Medium (DMEM; high glucose, no sodium pyruvate; Thermo Fisher Scientific) supplemented with 10% v/v fetal bovine serum (FBS; Sigma-Aldrich), 0.2 mM L-ascorbic acid 2-phosphate magnesium salt (Sigma-Aldrich), and 100 U mL^−1^ penicillin/streptomycin (Thermo Fisher Scientific).

Human mesenchymal stromal cells (hMSCs) were isolated as previously described from bone marrow aspirate of a donor [25] who had given consent based on approval from the Medical Ethical Committee of the Medisch Spectrum Twente hospital, Enschede, The Netherlands (study protocol ‘Functioneel weefselherstel met behulp vanuit beenmerg verkregen stamcellen’; K06-002). After isolation, the cells were seeded at a density of 1500 cells cm^−2^ in T-flasks and expanded in hMSC basic cell culture medium (BM_B_), consisting of minimum essential medium (MEM) α without nucleotides and with GlutaMAX (Thermo Fisher Scientific) and supplemented with 10% v/v FBS, 0.2 mM L-ascorbic acid 2-phosphate magnesium salt, and 100 U mL^−1^ penicillin/streptomycin.

The cells were cultured in standard conditions, in an incubator at 37 °C in a humidified atmosphere with 5% carbon dioxide (CO_2_). The medium was replaced every 2–3 days and the cells were passaged when reaching 70–80% confluence. The cells used in this study were at passages 3–4.

### 2.2 Fabrication, characterization, and preparation of polycarbonate microwell arrays

The U-bottom (circular) microwells were fabricated by micro pressure (thermo)forming [26] of an polycarbonate (PC) film produced in-house with a thickness of 50 μm, resulting in 289 microwells arranged in a honeycomb-like fashion (Supplementary Fig. 1). The polymer film was first heated to 153 °C to soften it and subsequently stretched into the microcavities of a brass mold using nitrogen at a pressure of 15 bar (Supplementary Fig. 1A). After cooling the film, releasing the pressure, and demolding, the microwell arrays were punched out to fit into the bottoms of 24-well plates. The size of the microwells was chosen based on the dimensions of the human enthesis, which is approximately 500 μm in length [12]. The outer diameter and the depth of the microwells were 555.6 ± 5.2 μm and 352.5 ± 2.3 μm, respectively, as measured using a VK-200 confocal laser scanning microscopy-based optical profilometer (VK-200, Keyence; Supplementary Fig. 1B).

Prior to cell culture, the microwell arrays were wetted and sterilized in a graded series of isopropanol (VWR; 100%, 70%, 50%, 25%, and 10% v/v in water) and then washed twice with Dulbecco's phosphate buffered saline (PBS; Sigma-Aldrich). The sterilized arrays were placed in 24-well plates and secured at the bottom of each well using sterile O-rings (ERIKS). To prevent cell attachment, the microwell arrays were incubated with 350 μL of 1% w/v Pluronic F108 (Sigma-Aldrich) solution per array at 37 °C overnight. The Pluronic solution was removed, and the microwell arrays were washed three times with sterile PBS. The microwells were then ready for cell culture.

### 2.3 Spheroid generation and culture

Single-cell-type spheroids were formed by seeding either ACL cells in BM_L_ or hMSCs in BM_B_ into the microwell arrays at a density of approximately 500,000 cells per microwell array, standardizing the initial spheroid size. The cells were incubated at 37 °C under 5% CO_2_ for 48 h, during which the cells aggregated into spheroids. Then, the culture medium was gently refreshed to remove cells that did not incorporate into spheroids and replaced with the respective ligament or osteogenic differentiation media. The differentiated state of ACL-derived spheroids was maintained for 7 or 21 days in ACL differentiation medium (DM_L_) composed of BM_L_ supplemented with 0.01 μg mL^−1^ transforming growth factor (TGF)-β3 (Peprotech). Differentiation of hMSC-derived spheroids toward the osteogenic lineage was induced using BM_B_ supplemented with 10^−8^ M dexamethasone (Sigma) and 10^−2^ M β-glycerophosphate (Sigma) for 15 or 21 days. The cell aggregates were further cultured at 37 °C under 5% CO_2,_ and the medium was refreshed every 2–3 days.

### 2.4 Spheroid fusion into multicellular microtissues

To induce spheroid fusion, hMSC-derived osteogenic spheroids were collected from a microwell array at defined time points, after gently pipetting up and down multiple times. The suspension was collected in a 15 mL conical tube (Falcon) and centrifuged at 300 rpm for 4 min. The supernatant was then aspirated, and the spheroids were resuspended in 100 μL of BM_L_. The spheroid suspension was gently dispensed into a microwell array containing the ACL-like spheroids pre-cultured for the respective duration and allowed to settle at 37 °C for 1 h. Next, 1 mL of ACL differentiation medium was gently added to the culture and refreshed every other day.

### 2.5 Fabrication, characterization, and preparation of the agarose geometric milliwell array

Arrays of square ‘geometric’ agarose milliwells were fabricated in agarose hydrogel by replica molding (Supplementary Fig. 2A). For this, elastomeric stamps of poly(dimethylsiloxane) (PDMS; Sylgard 184, Dow Corning) were produced using a SU-8-silicon wafer-based lithographic master. After sterilization, the PDMS stamps were used to replicate the milliwell arrays in 4% agarose (ultra-pure; Invitrogen). Briefly, agarose solution was cast onto the PDMS stamps and demolded upon solidification, and punched out to fit into the bottom of a 6-well plate. The side length and depth of the square milliwells in the master structure were 1002 ± 14 μm and 436 ± 12 μm, respectively, as determined using a confocal laser scanning microscopy-based optical profilometer (VK-200, Keyence; Supplementary Fig. 2B).

The milliwell arrays were placed into the bottom of a 6-well plate. Prior to cell culture, the agarose milliwells were sterilized by immersion in 70% ethanol for 1 h. The ethanol was then allowed to evaporate for 10–15 min until the surface was free of visible liquid, but the agarose remained moist. After this, the arrays were washed twice with PBS.

### 2.6 Spheroid fusion in the agarose geometric array

The ACL and osteogenic spheroids were flushed out of five microwell arrays after the respective culture periods (see section 2.3.), collected by centrifugation, resuspended in fresh DM_L_, and seeded into a single geometric array at a density of 50–100 spheroids per square milliwell. To minimize variability, spheroid concentration and transfer volume were standardized across experiments. The medium was refreshed daily. After 3 days of culture, the fused minitissues were collected for downstream analysis.

### 2.7 Tissue fixation and cryosectioning

The minitissues fused in the milliwells of the geometric arrays were fixed in 4% w/v paraformaldehyde (PFA; Sigma-Aldrich) solution at room temperature (RT) for 20 min. The tissues were then embedded in freezing medium (15% w/v sucrose and 7.5% w/v gelatin in 1 M phosphate buffer) and pre-cooled on ice. Freezing was performed in liquid nitrogen, and the blocks were stored at −80 °C until cryosectioning at −18 °C into 14-μm-thick sections. The sections were stored at −30 °C until use.

### 2.8 Acoustic stimulation

The samples were acoustically stimulated using a custom-built bioreactor setup as an add-on device for standard 6-well plates (VWR) (Supplementary Fig. 3) [27]. It comprised a commercial sound source (Diaphragm External Piezo Buzzer, RS Components) and a customized well insert. The well insert was milled from a 15-mm-thick PMMA sheet (PerlaPlast) using a computer numerical control (CNC) milling machine (monoFab SRM-20, Roland DG). The acoustic stimulation was applied using a ramp function at a frequency of 3000 Hz and an amplitude of 12 V_PP_ for a duration of 4 h per day for seven consecutive days.

### 2.9 Viability staining

To assess spheroid cell viability, live/dead staining was performed according to the manufacturer's protocol. The spheroids were washed twice with PBS, then incubated for 30 min with the LIVE/DEAD Fixable Far-Red Dead Cell Stain Kit (Molecular Probes, Thermo Fisher Scientific). Next, the samples were washed twice with PBS and fixed in 4% w/v PFA at RT for 20 min. F-actin was labeled with phalloidin conjugated to Alexa Fluor 488 (1:100; Thermo Fisher Scientific), and cell nuclei were counterstained with 4′,6-diamidino-2-phenylindole (DAPI; 1:100; Sigma-Aldrich) at RT for 30 min. The samples were stored in PBS until imaging using a confocal laser scanning fluorescence microscope (TCS SP8 STED, Leica Microsystems). For all samples, z-stacks with 3-μm-thick slices were acquired.

### 2.10 Immunofluorescence staining and confocal fluorescence microscopy

The microtissues were fixed in 4% PFA at RT for 20 min, washed three times with PBS, and permeabilized with 0.1% v/v Triton X-100 (Acros Organics) at RT for 1 h. After three washing steps, non-specific binding sites were blocked in CAS-Block (Thermo Fisher Scientific) at RT for 2 h. The blocking solution was then removed, and the microtissues were incubated with the primary antibodies (Supplementary Table 1) diluted in CAS-Block solution at 4 °C overnight. The next day, the samples were washed three times with PBS and incubated with the secondary antibodies (Supplementary Table 2) at RT for 2 h. The osteogenic spheroids were stained with OsteoImage (OsteoImage Mineralization Assay, Lonza), according to the manufacturer's protocol, to visualize mineral deposits. Finally, the samples were counterstained with DAPI (Sigma-Aldrich) to visualize the cell nuclei and mounted with coverslips using Dako Fluorescence Mounting Medium (Agilent). All stained samples were imaged using a confocal laser scanning fluorescence microscope (TCS SP8 STED, Leica Microsystems). The images were acquired as z-stacks with 3-μm-thick optical sections at a 25 × magnification using a water immersion objective.

### 2.11 RNA isolation and gene expression analysis

Total RNA was isolated using the RNeasy Mini Kit (QIAGEN) according to the manufacturer's protocol. Next, RNA purity and concentration were measured using a micro-volume spectrophotometer (μLITE, BioDrop). Complementary DNA (cDNA) was synthesized using the iScript cDNA Synthesis Kit (Bio-Rad) from 200 ng of total RNA. Gene expression analysis was performed by quantitative reverse transcription PCR (RT-qPCR) using the iQ SYBR Green Supermix (Bio-Rad) in a qPCR instrument (CFX96 Real-Time PCR Detection System, Bio-Rad), with 20 ng of cDNA per reaction. Gene expression levels were quantified using the ΔΔCt method with glyceraldehyde 3-phosphate dehydrogenase (*Gapdh*) or 18S ribosomal RNA (*18S rRNA*) as housekeeping genes. Results are presented as relative gene expression normalized to control conditions. Primer sequences for each gene are listed in Supplementary Table 3.

### 2.12 Transmission electron microscopy

For transmission electron microscopy (TEM), the samples were fixed with fixative composed of 1.5% glutaraldehyde (Sigma) in 0.067 M cacodylate buffer (Sigma) at pH 7.4 containing 1% sucrose (Sigma). The samples were then washed in washing buffer (0.1 M cacodylate, pH 7.4, containing 1% sucrose) and postfixed with 1% osmium tetroxide in the same buffer containing 1.5% potassium ferrocyanide at 4 °C in the dark for 1 h. The samples were dehydrated in ethanol, infiltrated with Epon resin, embedded, and polymerized at 60 °C for 48 h. Ultrathin sections were obtained using an ultramicrotome (Leica Ultracut UCT, Leica Microsystems) and mounted on Formvar-coated copper grids. The sections were stained with 2% uranyl acetate in water and lead citrate. Then, the sections were analyzed using an electron microscope (Tecnai Spirit T12) equipped with a CCD camera (Eagle 4kx4k, Thermo Fisher Scientific).

### 2.13 Image analysis

To assess the size and shape of the single-cell type spheroids, multicellular microtissues, and tissues formed within the geometric arrays, we quantified their area and circularity from bright-field images acquired using an inverted microscope (CKX53, Olympus). Image analysis was performed in ImageJ (https://imagej.net/ij/). Each image was first converted to an 8-bit image and thresholded to obtain a binary image, after which area and circularity values were extracted using shape descriptors. Circularity was calculated as 4 × π × area/perimeter^2^. For bright-field-based measurements, spheroid area was quantified from a single 2D image acquired at the largest cross-sectional (equatorial) plane of each spheroid (*i.e.*, the focal plane yielding the maximal apparent diameter), using microscope-calibrated pixel size. Only fully visible, in-focus spheroids not touching image borders were included.

To analyze the fusion of spheroids into multicellular aggregates, bright-field images were collected at multiple time points and processed in ImageJ. The included angles at the fusion interface were measured using ImageJ's Angle tool, allowing us to track changes over time. In addition, the length of the merged region (*i.e.*, the contact length between two spheroids) was quantified using the Line tool in ImageJ.

Quantification of cell viability and ECM production was performed using customized pipelines in CellProfiler (version 4.2.0; https://cellprofiler.org/) [28]. Briefly, in each pipeline, nuclear morphology was identified as a primary object using the Otsu adaptive thresholding method on the DAPI channel; cell morphology was determined using the propagation algorithm combined with the Otsu adaptive thresholding on the phalloidin channel. Cells touching the edges of the images were excluded from the dataset. Dead cells were quantified as the pixel area stained by the dead cell marker relative to the total segmented cell area. For area quantification from fluorescence images or confocal z-stacks, the projected spheroid area was calculated from maximum-intensity projections of phalloidin-based segmentation outputs, using identical pixel calibration. After background correction, collagen type III (COL-III), decorin (DCN), COL-I, osteocalcin (OCN), OsteoImage, and COL-X staining was quantified as the pixel area stained by the respective marker relative to the total segmented cell area.

The overlap coefficient between the COL-X and either the mineral-associated or DCN image channel was quantified using the Colocalization module in CellProfiler. For each pair of images, the overlap coefficient *r* is defined as *r* = sum(*R*_*i*_ × *G*_*i*_)/sqrt(sum(*R*_*i*_^2^) × sum(*G*_*i*_^2^)), where *R*_*i*_ and *G*_*i*_ represent the pixel intensities in the COL-X images (*R*) and the mineral-associated or DCN channel (G), respectively; *r* ranges from 0 (no overlap) to 1 (perfect overlap).

### 2.14 Statistical analysis

In bar graphs, bars and error bars represent mean values and standard deviation (SD), respectively. All experiments were performed with at least three independent biological replicates. Statistical analyses were performed using Prism (GraphPad Software; version 11.0.0). Data normality was assessed prior to statistical testing.

## 3 Results and discussion

### 3.1 Spheroid culture in microwell arrays

We fabricated PC microwell arrays as a spheroid culture platform by microthermoforming (Supplementary Fig. 1) [26]. Previously, we used a similar method to fabricate elongated microwells to study the elongation of free-floating P19C5 mouse embryonal carcinoma stem cell aggregates [29], and microcurved porous cell culture substrates with a curvature radius of 100 μm in a three-dimensional (3D) lung-on-chip model [30]. In an earlier study, Kim and colleagues employed cylindrical microwells with a length of 800 μm and a diameter of 400 or 800 μm to monitor spheroid fusion [31]. Unlike that study, which used an agarose hydrogel-based platform with 35 U-bottom microcavities, we opted for PC as the material of choice. Its biocompatibility, transparency, and low autofluorescence allowed for high-quality light-microscopic imaging to monitor spheroid formation and fusion process effectively over time.

After fabricating the PC microwell arrays and applying a non-adherent Pluronic coating, we separately seeded ACL cells and hMSCs into the microwells and allowed them to self-assemble into spheroids. For generating ACL-derived spheroids, ACL cells isolated from human donors were preferred over MSCs differentiated into ligament cells due to the limitations of existing differentiation protocols to produce cells with all the characteristics of ligament cells in their native environment [32]. However, for generating bone-like spheroids, hMSCs were used. MSCs are multipotent progenitor cells capable of differentiating into osteogenic, chondrogenic, and adipogenic lineages [33]. The hMSC cell culture medium was supplemented with ascorbic acid, dexamethasone, and β-glycerophosphate, which is a standard protocol for inducing osteogenic differentiation and mineralization. Spheroids were cultured for up to 21 days and characterized at defined time points depending on the experiment.

### 3.2 Characterization of ACL-derived spheroids

**Fig. 2:**
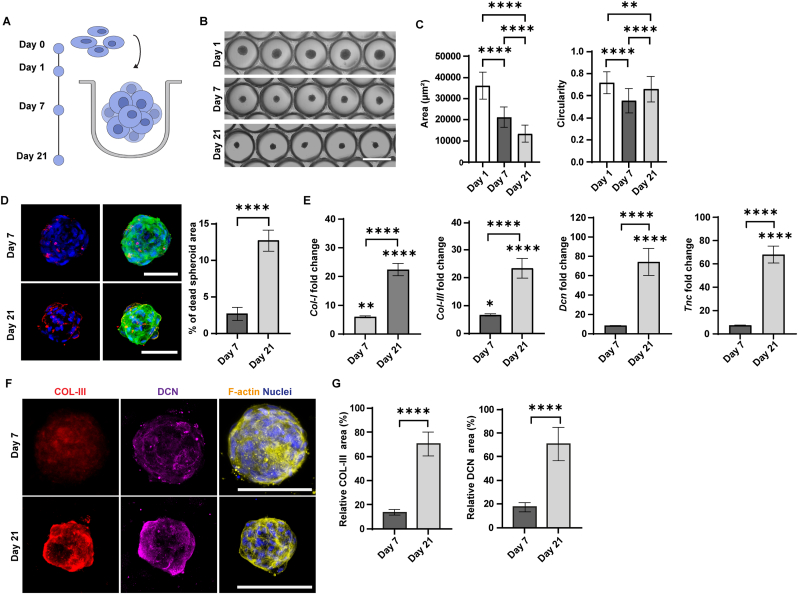
**Characterization of anterior cruciate ligament (ACL)-derived spheroids. A)** Illustration of ACL cell condensation and spheroid formation. **B)** Representative bright-field images showing different stages of ACL spheroid differentiation within microwell arrays. Scale bar represents 500 μm and applies to all images. **C)** Quantitative analysis of spheroid area and circularity after 1, 7, and 21 days of culture. Data were analyzed using the Brown-Forsythe test and Welch ANOVA, followed by Games-Howell post-hoc test for multiple comparisons. ∗∗p < 0.01, ∗∗∗∗p < 0.0001. N = 20. **D)** Evaluation of cell viability in ACL spheroids. Maximum intensity projections of confocal fluorescence microscopy images after 7 days (top) and 21 days (bottom) of culture. Cells are stained with LIVE/DEAD Fixable Far Red Dead Cell Stain (red) to label the dead cells, DAPI (blue) to visualize cell nuclei, and phalloidin (green) to label F-actin. Scale bars represent 100 μm and apply to all images in the same row. Bar graph showing the quantification of the percentage of dead cells in ACL spheroids after 7 and 21 days of culture. Normality was assessed by the Shapiro-Wilk test, and group differences were tested using Welch's *t*-test. ∗∗∗∗p < 0.0001. N = 10. **E)** Bar graphs showing the expression of ACL markers at mRNA level. Values are given as fold changes relative to day 1. Normality was assessed by the Shapiro-Wilk test, and group differences were tested using Welch's *t*-test. ∗p < 0.05, ∗∗p < 0.01, ∗∗∗∗p < 0.0001. Asterisks directly above the bars indicate comparison to day 1. N = 3. **F)** Confocal fluorescence images showing extracellular matrix component distribution in ACL spheroids on day 7 (top) and day 21 (bottom) of culture. Spheroids were labeled for collagen type III (COL-III; red), decorin (DCN; purple), F-actin (yellow), and cell nuclei (blue). Scale bars represent 100 μm and apply to all images in the same row. **G)** Quantification of matrix component area relative to total spheroid area on days 7 and 21 of culture. Normality was assessed by the Shapiro-Wilk test, and group differences were tested using Welch's *t*-test. ∗∗∗∗p < 0.0001. N = 10. (For interpretation of the references to color in this figure legend, the reader is referred to the Web version of this article.)

ACL cells self-assembled into spheroids of uniform size within 24 h. First, we assessed the dimensions of the ACL-derived spheroids on days 1, 7, and 21 of culture (Fig. 2A). The average projection area of spheroids decreased significantly from approximately 35,000 ± 643 μm^2^ on day 1 by approximately 1.7-fold on day 7 and 2.6-fold on day 21 (Fig. 2B and C). This decrease in size has been previously reported in ACL spheroids during culture [34], and it is not solely due to cell death, as the cell viability within the spheroids remained above 85% over time (Fig. 2D). The size reduction likely results from increased cell-cell interactions leading to greater compaction. Circularity values remained relatively stable across all time points, and the spheroids maintained a compact, round shape after day 1 (Fig. 2B and C).

To evaluate the maturation of ACL spheroids, we analyzed the expression of key ligament ECM components at the gene level on days 1, 7, and 21 (Fig. 2E). The expression of collagen type I (*Col-I*) and type III (*Col-III*) progressively increased during the culture period. Specifically, *Col-I* was upregulated 5.3-fold on day 7 relative to day 1 and 4.4-fold on day 21 compared to day 7, resulting in a total increase of 23-fold from day 1 to day 21. *Col-III* showed an approximately 7-fold increase on day 7 compared to day 1 and a 3-fold increase on day 21 relative to day 7, with a total increase of 23-fold from day 1 to day 21. The expression levels of decorin (*Dcn*) and tenascin-C (*Tnc*) were enhanced on day 7 compared to day 1 (7.3- and 7.8-fold increase, respectively), but significant upregulation compared to day 1 was observed only on day 21. On day 21, *Dcn* exhibited an approximately 10-fold increase compared to day 7, and *Tnc* showed an approximately 9-fold increase. Moreover, *Dcn* expression levels showed a significant 74-fold increase on day 21 compared to day 1, while *Tnc* demonstrated a significant 68-fold increase over the culture period.

At the protein level, we detected COL-III at both time points, but its expression and distribution were higher and more homogeneous on day 21 compared to day 7 (Fig. 2F and G and Supplementary Fig. 4). Additionally, DCN showed a significant 4-fold increase in deposition on day 21 relative to day 7.

The increased DCN expression at both the protein and gene levels is consistent with previous reports showing that DCN peaks during tendon maturation [35]. This alignment with earlier findings reinforces its role as a marker of active tissue development. During maturation, DCN plays a crucial role in collagen fibrillogenesis, contributing to the alignment and stabilization of fibrils [36]. Therefore, the progressive increase in DCN expression over the 21-day culture period likely reflects ongoing matrix production and tissue maturation, which could explain the overall enhanced expression of ACL markers observed at the final time point. In conclusion, the employed microwell system supported the formation of ACL spheroids and their maturation over a 21-day culture period.

### 3.3 Characterization of hMSC-derived spheroids

**Fig. 3:**
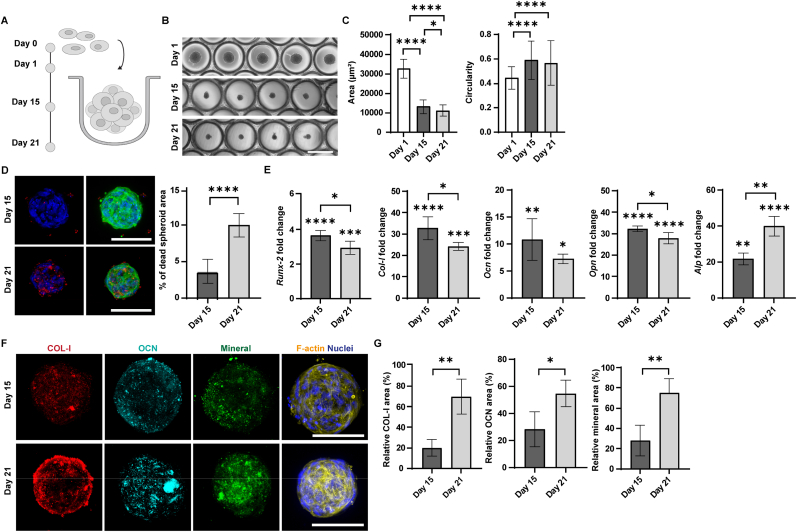
**Characterization of osteogenically differentiated human mesenchymal stromal cell (hMSC)-derived spheroids. A)** Illustration of hMSC condensation and subsequent osteogenic spheroid formation. **B)** Representative bright-field images showing different stages of hMSC spheroid differentiation within microwell arrays. Scale bar represents 500 μm and applies to all images. **C)** Quantitative analysis of spheroid area and circularity after 1, 15, and 21 days of culture. Data were analyzed using the Brown-Forsythe test and Welch ANOVA, followed by Games-Howell post-hoc test for multiple comparisons. ∗p < 0.05, ∗∗∗∗p < 0.0001. N = 20. **D)** Evaluation of cell viability in hMSC spheroids. Maximum intensity projections of confocal fluorescence microscopy images after 15 days (top) and 21 days (bottom) of culture. Cells are stained with LIVE/DEAD Fixable Far Red Dead Cell Stain (red) to label the dead cells, DAPI (blue) to visualize cell nuclei, and phalloidin (green) to label F-actin. Scale bars represent 100 μm and apply to all images in the same row. Bar graph showing the quantification of the percentage of dead cells in bone spheroids after 15 and 21 days of culture. Normality was assessed by the Shapiro-Wilk test, and group differences were tested using Welch's *t*-test. ∗∗∗∗p < 0.0001. N = 10. **E)** Bar graphs showing the expression of osteogenic markers at mRNA level. Values are given as fold changes relative to day 1. Normality was assessed by the Shapiro-Wilk test, and group differences were tested using Welch's *t*-test. ∗p < 0.05, ∗∗p < 0.01, ∗∗∗p < 0.001, ∗∗∗∗p < 0.0001. Asterisks directly above the bars indicate comparison to day 1. N = 3. **F)** Confocal fluorescence images showing extracellular matrix component distribution in osteogenic spheroids on day 15 (top) and day 21 (bottom). Spheroids are labeled for collagen type I (COL-I; red), osteocalcin (OCN; cyan), mineral (green), F-actin (yellow), and cell nuclei (blue). Scale bars represent 100 μm and apply to all images in the same row. **G)** Quantification of matrix component area relative to total spheroid area after 15 and 21 days of culture. Normality was assessed by the Shapiro-Wilk test, and group differences were tested using Welch's *t*-test. ∗p < 0.05, ∗∗p < 0.01. N = 10. (For interpretation of the references to color in this figure legend, the reader is referred to the Web version of this article.)

Similar to the ACL cells, hMSCs also formed spheroids of uniform size within 24 h. We characterized hMSC-derived spheroids at different time points (days 1, 15, and 21) during the culture period (Fig. 3A). The spheroid projection area decreased significantly from approximately 35,000 ± 973 μm^2^ on day 1 to 17,000 ± 258 μm^2^ on day 15 and to 14,000 ± 251 μm^2^ on day 21 (Fig. 3B and C), consistent with our previous findings that hMSC spheroids compact over time [37,38]. Circularity values increased from 0.45 on day 1 to 0.6 and 0.58 on days 15 and 21, respectively. Dead-cell staining was detectable at day 15 but remained limited, below 5% of the total spheroid area, and increased by day 21 to approximately 10% of the total area, corresponding to approximately 90% viability (Fig. 3D).

Next, we monitored the maturation of the aggregates over time. Osteoprogenitor cells can differentiate into pre-osteoblasts, which are characterized by the expression of early markers of osteogenic differentiation, such as runt-related transcription factor 2 (*Runx2*), the ‘master gene’ for osteogenesis, and COL-I [39], the main matrix protein in bone. At the gene expression level, *Runx2* showed a 3.8-fold upregulation on day 15 compared to day 1 (Fig. 3E), followed by a slight decrease on day 21, resulting in an overall 3-fold increase from day 1 to day 21. *Col-I* exhibited a 33-fold upregulation at day 15, followed by a slight decline at day 21, yielding in a overall 24-fold increase from day 1 to day 21. When pre-osteoblasts differentiate into fully functional osteoblasts, an increase in the expression of osteocalcin (*Ocn*) can be observed. As a late osteoblast differentiation marker, it has also been reported to participate in bone-to-organ endocrine crosstalk [39,40]. qPCR data confirmed a significant upregulation of *Ocn* throughout the culture period, with a 10-fold increase on day 15 and a 7.2-fold increase on day 21 compared to day 1. We also measured the expression of the SPP1 (secreted phosphoprotein 1) gene, in this article denoted as *Opn*, which encodes the matrix-associated protein osteopontin (OPN). OPN is known as a regulator of mineralization. Its functions include local restriction of hydroxyapatite growth and enzymatic control of inhibitory matrix components, for example, phosphate-regulating endopeptidase homolog, X-linked (PHEX)-dependent processing, as conceptualized in the “stenciling principle” framework [39,[41], [42], [43], [44]]. *Opn* expression levels were significantly increased, with a 32-fold increase on day 15 and a 28-fold increase on day 21 compared to day 1. Furthermore, alkaline phosphatase (*Alp*), a gene encoding an enzyme that promotes mineralization in part by hydrolysing inorganic pyrophosphate (PPi), a potent inhibitor of hydroxyapatite formation, thereby supporting initiation and propagation of bone mineral deposition [45,46], displayed significant upregulation. A 20-fold increase was observed on day 15 and a 40-fold increase on day 21 compared to day 1.

At the protein level (Fig. 3F and G), immunostaining confirmed the expression of COL-I, which significantly increased on day 21 compared to day 15. Similarly, on day 21, the spheroid area stained positively for OCN, which showed a significant increase relative to day 15. Fully mature osteoblasts are characterized by their ability to synthesize osteoid, which becomes mineralized by the formation of hydroxyapatite [47]. Staining and quantification of mineral deposits on days 15 and 21 demonstrated approximately a 45% increase in the spheroid area covered by hydroxyapatite deposits on day 21 compared to day 15. Notably, the modest decrease in *Col-I* and *Ocn* mRNA from day 15 to day 21 coincides with increased COL-I and OCN immunostaining, consistent with osteogenic cultures progressing toward matrix maturation/mineralization. Transcript levels may plateau or decline, while secreted matrix proteins are retained and continue to accumulate in the ECM. In conclusion, the microwell platform proves to be a valuable culture system for generating osteogenic spheroids from hMSCs, as demonstrated by our assessment of spheroid size, shape, and expression of key osteogenic markers.

### 3.4 Fusion of ligament and bone spheroids into multicellular microtissues

The ACL-derived and hMSC-derived spheroids, hereafter referred to as ligament- (L) and bone-like spheroids (B), respectively, were induced to form multicellular aggregates through fusion by adjacent co-culturing in the microwell arrays. We used spheroid fusion rather than mixed-cell co-aggregation because it allows each compartment to undergo independent differentiation and maturation, including ECM preconditioning, over distinct time courses prior to assembly. Previous studies have used various techniques to induce the fusion of heterotypic spheroids, such as programmable merging of adjacent droplets [48] or using elongated cavities and 3D-scaffold frameworks to force spheroid fusion in linear arrays [31,49,50].

**Fig. 4:**
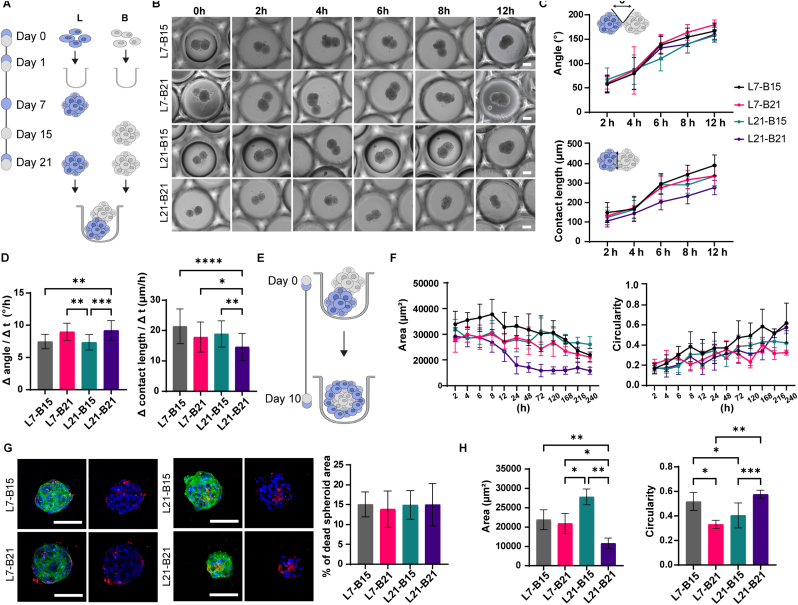
**Dynamics of spheroid fusion within the microwell system. A)** Schematic representation of the experimental timeline for spheroid fusion. **B)** Bright-field microscopy images showing the time course of the fusion process over a 12 h period after co-seeding. Each pair consisted of ligament (L) and bone spheroids (B) of different maturity (7, 15, or 21 days). Scale bars represent 100 μm and apply to all images in the same row. **C)** Quantification of the tangent angles (top) and contact length (bottom) at the fusion points between the merged spheroid pairs over time. N = 20. **D)** Fusion kinetics represented by the rate of angle change at 12 h post-co-seeding for the four different spheroid pairs. Data represent mean ± SD. Data were analyzed using the Kruskal-Wallis test, followed by Dunn's post hoc test for multiple comparisons. ∗p < 0.05, ∗∗p < 0.01, ∗∗∗p < 0.001, ∗∗∗∗p < 0.0001. N = 20. **E)** Schematic representation of the experimental timeline following spheroid fusion. **F)** Quantification of aggregate area (left) and circularity (right) over 10 days of culture. N = 20. **G)** Assessment of cell viability in multicellular aggregates after 10 days of culture. Maximum intensity projections of confocal fluorescence microscopy images stained with LIVE/DEAD Fixable Far Red Dead Cell Stain (red) to label the dead cells, DAPI (blue) to visualize cell nuclei, and phalloidin (green) to label F-actin. Scale bars represent 100 μm and apply to all images in the same row. Bar graph showing the quantification of the percentage of dead cells in the fused spheroids. Data were analyzed using the Kruskal-Wallis test, followed by Dunn's post hoc test for multiple comparisons. N = 10. **H)** Bar graphs showing multicellular aggregates' area (left) and circularity (right) after 10 days of culture. Data were analyzed using the Kruskal-Wallis test, followed by Dunn's post hoc test for multiple comparisons. ∗p < 0.05, ∗∗p < 0.01, ∗∗∗p < 0.001. N = 20. (For interpretation of the references to color in this figure legend, the reader is referred to the Web version of this article.)

The maturation stage at which the discrete spheroids undergo fusion has been shown to influence the fusion kinetics and properties of the resulting microtissue [50]. To investigate this, we pre-cultured ACL spheroids for either 7 or 21 days, as single spheroid analysis indicated progressive maturation over time. Similarly, hMSC spheroids were pre-differentiated for either 15 or 21 days. After the pre-culture period, the spheroids were harvested and co-seeded in the microwell arrays, resulting in four distinct maturity combinations designated according to the spheroids’ pre-culture durations: L7-B15, L7-B21, L21-B15, and L21-B21 (Fig. 4A).

We monitored the spheroid fusion by microscopy at 2, 4, 6, 8, and 12 h after seeding (Fig. 4B). The fusion process was described by measuring the tangent angles and contact length at the fusion points between the merged spheroids. In general, the tangent angle increased over time for all spheroid pairs (Fig. 4C and Supplementary Fig. 5A). Throughout 12 h, the average angle between L7-B15 spheroid pairs increased from 57.95 ± 18.30° to 166.91 ± 16.68°, corresponding to a rate of angle change (Δ angle/Δ t, here referred to as fusion rate) of 7.57 ± 1.16° h^−1^ (Fig. 4D). In comparison to L7-B15, the fusion kinetics were not influenced by the incorporation of more mature bone spheroids in L7-B21 spheroid pairs, as evidenced by a similar fusion rate of 8.8° h^−1^. The presence of more mature ligament spheroids (L21) in combination with B15 aggregates slightly slowed down the fusion process at later time points, leading to a fusion rate of 7.35° h^−1^. A significantly higher angle change rate of 9.19° h^−1^ was observed for L21-B21 spheroid pairs compared to groups containing less mature bone spheroids (B15). These findings indicate the impact of the maturation stage of spheroids on fusion kinetics. Previous observations have shown that longer pre-culture times of spheroids lead to lower fusion rates [50]. However, this pattern did not apply to the more mature bone spheroids in our system. We hypothesize that the size of the bone spheroids influences fusion kinetics, with the smaller B21 spheroids leading to a higher rate of angle change. To better understand this process, it would be interesting to evaluate and compare the fusion rates of spheroids at different maturation stages while maintaining equivalent size. By adjusting the initial cell seeding density, it may be possible to predict and control the size of the aggregate at different time points before fusion.

The contact length, which represents the merged region between two spheroids, gradually increased over time (Fig. 4C and Supplementary Fig. 5B). Consistent with the smaller size of mature single spheroids, L21-B21 pairs showed a shorter contact length compared to the other experimental groups from 6 to 12 h post-co-seeding. At the final time point, L21-B21 pairs demonstrated a 2.6-fold decrease in contact length compared to L7-B15 pairs, a 1.9-fold decrease compared to L21-B15, and a 1.2-fold decrease relative to L7-B21 pairs. The rate of change in contact length over 12 h was also significantly lower in L21-B21 spheroid pairs compared to the other conditions (Fig. 4D). The fold decrease ranged from 1.2 (compared to L7-B15) to 1.5 (compared to L21-B15). In conclusion, our findings further confirmed the impact of spheroid maturation on fusion kinetics, highlighting the importance of spheroid size and maturation for the fusion and integration of bone-ligament-like interfaces.

Upon fusion, tissue-like structures remodel, deform, and stabilize their shape [37]. Therefore, we evaluated the changes in size and shape of the aggregated spheroids during the fusion process, starting from 2 h post-co-seeding and continuing for 10 days (Fig. 4E). Bright-field images of the 3D assembled constructs showed a consistent decrease in the aggregates' projection area across all the different conditions (Fig. 4F). Specifically, L7-B15, L7-B21, and L21-B15 aggregates initially formed with an area of approximately 30,000–35,000 μm^2^ at 2 h post-co-seeding. The area progressively decreased over time, reaching below 30,000 μm^2^ on day 10. L21-B21 aggregates also showed an initial area of approximately 30,000 μm^2^ at 2 h post-co-seeding, which decreased over time and ultimately reached an area of approximately 18,000 μm^2^ on day 10. L21-B21 aggregates consistently exhibited the smallest areas compared to the other groups at all analyzed time points.

To characterize the morphology of the aggregates, we measured their circularity values at different time points (Fig. 4F). Initially, at 2 h after co-seeding, the circularity values ranged between 0.14 and 0.25, gradually increasing to 0.3–0.6 over time. In L7-B15 aggregates, there was an increase in circularity compared to both L21-B15 (1.5-fold at 6 h) and L7-B21 (1.2-fold at 6 h). This trend persisted on days 3 and 5 post-co-seeding. By day 10, L7-B21 exhibited the lowest circularity value of 0.32, nearly half of that observed in L7-B15 and L21-B21. This suggests that the maturation stage of the initial spheroids influences the fusion efficiency and morphology of the resulting aggregates. In conclusion, the concomitant decrease in the projection area of multicellular aggregates, along with the increase in circularity values, indicates the formation of compact multicellular aggregates over 10 days. These changes suggest that as the aggregates mature, they become more spherical.

Next, we characterized the aggregates at 10 days post-co-seeding. First, we evaluated the viability of the multicellular aggregates, calculated as the ratio of dead cell area to total aggregate area (Fig. 4G). For all four combinations of fused spheroid pairs, viability remained higher than 85%, without significant differences between the different groups. The incorporation of lineage-resolved approaches, such as reporter systems prior to fusion and single-cell or spatial profiling, could help quantify compartment-specific proliferation and survival within the fused microtissues.

The morphological parameters of the aggregates, including size and circularity, were quantified in fluorescence microscopy images on day 10 (Fig. 4H). Similar to the bright-field imaging results, the projection area was significantly smaller in mature L21-B21 aggregates compared to the other three pairs, while L21-B15 pairs displayed the highest values of projection area among all the groups. The circularity values varied across the different groups. The early-stage (L7-B15) and late-stage (L21-B21) aggregates had circularity values ranging between 0.5 and 0.6, which were significantly higher compared to the other two groups, with circularity values ranging between 0.33 and 0.40. In conclusion, after 10 days of co-seeding, the spheroids remained viable and exhibited variable sizes between spheroid pairs but similar round shapes.

### 3.5 Characterization of multicellular microtissues

**Fig. 5:**
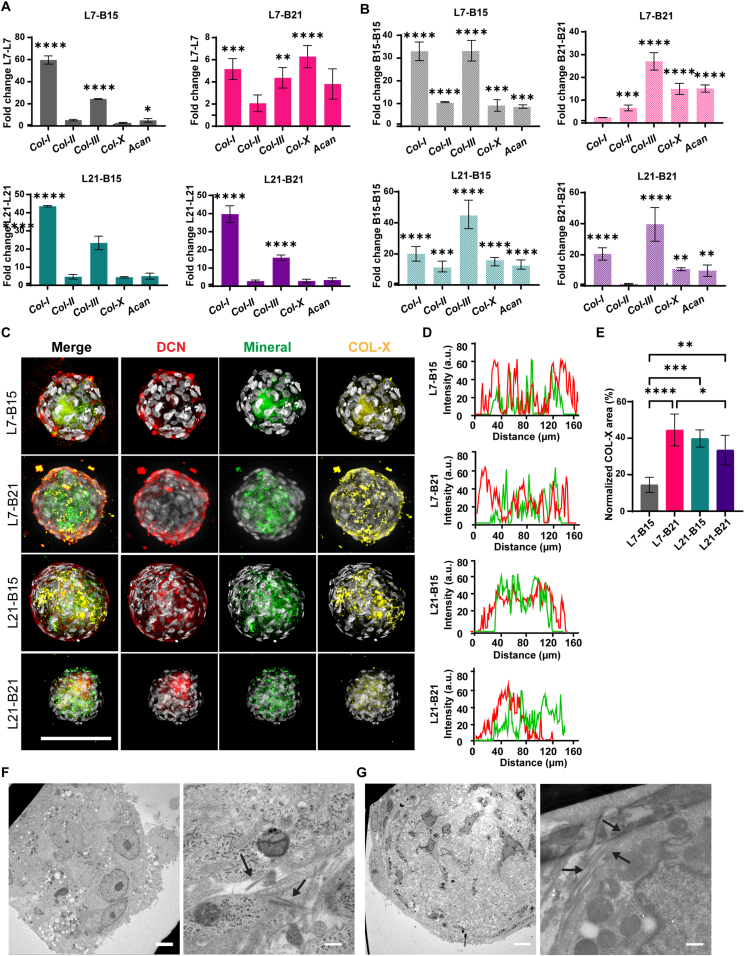
**Characterization of spheroid fusion-derived microtissues relevant to enthesis repair. A** and **B)** Bar graphs showing the expression of the enthesis-related genes collagen type I (*Col-I*), type II (*Col-II*), type III (*Col-III*), type X (*Col-X*)*,* and aggrecan (*Acan*) in L7-B15 (gray), L7-B21 (pink), L21-B15 (green), and L21-B21 (purple) microtissues. Values are presented as fold changes relative to (A) fused ligament spheroids (L7-L7 or L21-L21) or (B) fused bone spheroids (B15-B15 or B21-B21) of the same maturity stage. Data were analyzed using the Kruskal-Wallis test, followed by Dunn's post hoc test for multiple comparisons. ∗p < 0.05, ∗∗p < 0.01, ∗∗∗p < 0.001, ∗∗∗∗p < 0.0001. N = 3 **C)** Maximum intensity projections of confocal fluorescence microscopy images of multicellular microtissues cultured for 10 days and labeled for decorin (DCN; red), mineral (green), and collagen type X (COL-X; yellow), and counterstained with DAPI to visualize cell nuclei (gray). Scale bar represents 100 μm and applies to all images. **D)** Fluorescence intensity profiles showing the distribution of DCN (red) and mineral (green) across (from top to bottom) L7-B15, L7-B21, L21-B15, and L21-B21 aggregates. Fluorescence intensity is shown in arbitrary units (a.u.). **E)** Bar graphs showing COL-X quantification in the multicellular aggregates. Data were analyzed using the Kruskal-Wallis test, followed by Dunn's post hoc test for multiple comparisons. ∗p < 0.05, ∗∗p < 0.01, ∗∗∗p < 0.001, ∗∗∗∗p < 0.0001. N = 10. **F** and **G)** Transmission electron microscopy images of (F) L7-B15 and (G) L7-B21 aggregates. In each pair of images, the left one is the overview image of the microtissue, and the right one is the magnified image of the merging region with the arrows indicating collagen fibers. Scale bars represent 5 μm (left images) and 500 nm (right images). (For interpretation of the references to color in this figure legend, the reader is referred to the Web version of this article.)

The direct interaction between cells derived from tendons or ligaments (*e.g.*, fibroblasts) and bone (*e.g.*, osteoblasts) plays a key role in fibrocartilage formation and the expression of interface markers found in the enthesis [19]. Therefore, we first examined the expression of relevant enthesis-related genes, including *Col-I*, *Col-III*, collagen X (*Col-X*), and aggrecan (*Acan)* [3,5,12,15], within the multicellular microtissues (Fig. 5A and B). Gene expression was quantified as fold change compared to their respective constituent spheroids, *i.e.*, fused homotypic ligament (L-L) or bone spheroids (B-B) at the same maturation stage, after 10 days of merged culture. In L7-B15 aggregates, *Col-I* expression increased 60-fold compared to the L7-L7 control and 32-fold compared to the B15-B15 control. *Col-III* expression showed 25-fold and 30-fold increases compared to L7-L7 and B15-B15 controls, respectively. Additionally, collagen type II (*Col-II*) and *Col-X* were upregulated 10-fold and 11-fold compared to B15-B15 controls, while *Acan* was upregulated approximately 10-fold relative to both controls. When more mature bone spheroids were incorporated in the fused microtissues, *i.e.*, in L7-B21 aggregates, we measured a 5-fold increase in *Col-I* and *Col-III* expression, and a 6-fold increase in *Col-X* mRNA levels compared to L7-L7 spheroids. There was also a 7-fold upregulation of *Col-II*, 27-fold upregulation of *Col-III*, and 15-fold upregulation of *Col-X* and *Acan* compared to B21-B21 controls. In L21-B15 and L21-B21 aggregates, there was an approximately 40-fold increase in *Col-I* expression and an approximately 25- and 15-fold increase, respectively, in *Col-III* compared to L21-L21 controls. All markers were significantly upregulated in these aggregates compared to homotypic fused bone-like controls, except for *Col-II* in L21-B21 microtissues. In summary, in all fused microtissues, increased expression of the enthesis markers (or a subset of them) compared to controls was detected. It was previously reported that integrated layers of cell sheets comprising tenogenic and osteogenic progenitor cells showed significant expression of fibrocartilage markers compared to stacked cell sheets in growth medium but not compared to tenogenic-only culture [51]. In our case, the distinct expression patterns of enthesis-related genes in the multicellular aggregates may have been influenced by the maturation stage of the starting single-cell type spheroids (Supplementary Fig. 6). For instance, L7-B21 aggregates showed higher *Col-X* expression compared to L7-B15 aggregates, when normalized to their common L-L control (Supplementary Fig. 6C). *Col-X*, associated with hypertrophic chondrocyte differentiation [52], plays a crucial role in forming a transitional zone between the tendon or ligament and bone [53]. Conversely, *Col-I* expression was significantly downregulated in L7-B21 and L21-B15 aggregates compared to L7-B15 aggregates (Supplementary Fig. 6A and C). This likely reflects the dynamic remodeling during enthesis maturation, when cells at the insertion site exhibit a maturation gradient in collagen composition, progressing from COL-I toward COL-II and ultimately COL-X as mineralized fibrocartilage develops [4]. Importantly, COL-X becomes enriched and associated with hypertrophic or mineralizing fibrochondrocytes near the mineralization front [54]. At the same time, COL-I remains a major structural collagen in adjacent soft and hard tissue regions where COL-I synthesized by fibrochondrocytes at the insertion site is successively replaced by COL-X. Taken together, the differential expression patterns of *Col-I* and *Col-X* between L7-B15 and L7-B21 aggregates may suggest that the inclusion of late-stage bone spheroids contributed to a more advanced maturation stage of the enthesis microenvironment. This is consistent with a more advanced mineralizing fibrocartilage-like phenotype under our culture conditions. Building on this, our platform provides a controlled framework to implement lineage-resolved and region-specific readouts of enthesis maturation. In future studies, we will expand the marker panel to quantify maturation gradients across the junction, spanning from fibroblasts (predominantly characterized by *Col-I*/COL-I expression), to unmineralized fibrochondrocytes (*Col-I*/COL-I, *Col-II*/COL-II, and GLI family zinc finger 1, *Gli1*/GLI1), to mineralizing fibrochondrocytes (Indian Hedgehog, *Ihh*/IHH), and finally to mineralized fibrochondrocytes (*Col-X*/COL-X) [53]. Such a marker-based spatial profiling strategy would enable more efficient evaluation of whether engineered constructs progress toward a mature, zonally organized enthesis phenotype and would provide a tractable readout for testing pathway perturbations, such as Notch- and Hedgehog-related cues.

We also analyzed whether the expression of markers related to ligament or bone was affected by the fusion process (Supplementary Fig. 7). *Dcn* expression levels were comparable between all groups, while *Tnc* expression was downregulated relative to fused homotypic ACL spheroid pairs, with significant differences observed in L7-B15 and L21-B21 aggregates (Supplementary Fig. 7A). *Runx2* was significantly upregulated in all aggregates compared to the respective fused osteogenic spheroids (Supplementary Fig. 7B). In line with our findings, a previous study observed *Runx2* enrichment in cells at the interface [15]. *Alp* showed a significant 4-fold upregulation in L7-B15 aggregates, while its expression remained similar in other aggregates compared to osteogenic spheroids at the same maturation stage. *Ocn* and *Opn* expression levels were similar between the different groups and their controls. In conclusion, the fusion process modestly affected the expression of specific ligament- and bone-related markers, which may indicate their potential roles in enhancing the integration and maturation of the enthesis microenvironment.

To investigate the formation of a transitional zone between the ligament- and bone-like components within the multicellular aggregates, we visualized the organic ECM of ligament spheroids by immunofluorescence labeling of DCN and the mineral deposits in the osteogenic spheroids by OsteoImage staining (Fig. 5C). We did not include COL-I immunostaining of these fused constructs because COL-I is abundantly produced by both ligament and osteogenic spheroids and therefore provides limited spatial contrast for delineating compartmentalization or the emerging interface. Maximum intensity projection of confocal images showed that the interior structure of the fused L7-B15, L7-B21 and L21-B15 spheroids was compartmentalized such that a ligament-like shell fully surrounded a bone-like core. This core-shell-like organization is also evident in central optical sections of confocal z-stacks (Supplementary Fig. 8). The fluorescence intensity profile confirmed that the two-colored signals (red: ACL-like region; green: bone-like region) overlapped across the fused aggregates, with the mineral signal (green) mostly localized in the central area (Fig. 5D). This observation suggests that the ligament fibroblasts are capable of engulfing the osteogenic spheroids, independent of the size of the latter. In contrast, L21-B21 assemblies showed a different pattern, with the two spheroids mostly positioned at opposite sides of the aggregate.

The core-shell structures observed in L7-B15, L7-B21, and L21-B15 multicellular aggregates may result from ACL cells migrating over the mineral-rich, dense structure of bone spheroids. Similar engulfment phenomena have been previously described in other cell types, such as retinal progenitor cells enveloping spheroids of limbal MSCs in culture [55], and hepatic cells engulfing mesenchymal spheroids [56]. These observations indicate a potential mechanism driven by differential adhesion between cell populations, as proposed by the differential adhesion hypothesis [57]. According to this hypothesis, tissues or cell populations with higher apparent surface tension tend to be surrounded by those with lower surface tension [58]. The enhanced expression of adhesion molecules, such as cadherins [59], could lead to an increase in tissue surface tension by promoting cell-cell contacts. To better understand the mechanisms driving fusion and zonal organization, future studies should focus on characterizing the expression levels of adhesion molecules within the multicellular aggregates.

To analyze the formation of a transitional zone within the multicellular aggregates, we assessed COL-X expression and localization by immunofluorescence staining. Quantification of COL-X showed that L7-B15 aggregates had the lowest area occupied by COL-X deposits compared to the other groups (Fig. 5E). Specifically, L7-B21 aggregates exhibited a significant 3-fold increase in COL-X area compared to L7-B15, while L21-B15 aggregates showed a 2.7-fold increase and L21-B21 aggregates a 2.3-fold increase. The larger COL-X deposits in L7-B21, L21-B15 and L21-B21 aggregates indicate a more pronounced formation of the transitional zone between the ligament- and bone-like regions. Because COL-II is a canonical fibrocartilage marker in native entheses, we also evaluated COL-II immunostaining. However, under our current culture conditions, COL-II did not display a clearly interface-restricted pattern and instead appeared diffuse, limiting its value as a spatial readout of a discrete fibrocartilage-like zone (Supplementary Fig. 9). We therefore focused on COL-X localization as an interface-associated marker of mineralizing/hypertrophic fibrocartilage to quantify transitional zone formation.

To evaluate the spatial distribution of COL-X deposits within the multicellular aggregates, we quantified the degree of overlap between COL-X and DCN, as an indicator of ACL-like matrix, and between COL-X and mineral, as an indicator of bone-like matrix (Supplementary Fig. 10). Overlaps of COL-X with DCN and COL-X with mineral were assessed by measuring the percentage of co-localized pixels between the respective signals, with higher overlap percentages indicating greater spatial association. Overlaps of COL-X with ACL- and bone-like matrices were observed in all conditions, confirming the supportive role of COL-X in the establishment of a transitional zone between the ligament- and bone-like region. In addition, L7-B21 and L21-B15 aggregates showed a significantly higher degree of overlap between COL-X and DCN compared to COL-X and OsteoImage signal, suggesting higher co-localization of COL-X within the ACL-like matrix. In other conditions, overlap values between ACL- and bone-like matrices were comparable.

TEM analysis of L7-B15 and L7-B21 aggregates confirmed the presence of a distinct core-shell configuration (Fig. 5F and G, in each case left panel). In this configuration, a ring-like region containing large cells appeared to surround the central region, in which the mineral, as small dark dots, could be observed. In addition, in L7-B15 we observed the formation of short and immature collagen fibrils, while L7-B21 aggregates exhibited elongated and aligned collagen fibrils with distinct 67-nm D-periodicity in the merging region, suggesting a more advanced stage of collagen maturation (Fig. 5F and G, in each case right panel).

In conclusion, our results suggest that the formation of multicellular microtissues promotes the expression of interface-related characteristics of the mineralized fibrocartilage-like region of the enthesis model. These aggregates also maintain the zonal expression of ligament- and osteogenic-related markers, suggesting that our model recapitulates key aspects of the *in vivo* enthesis microenvironment. This model could therefore contribute to increasing the understanding of musculoskeletal tissue interfaces and may improve regenerative therapies and tissue engineering approaches.

### 3.6 Multicellular microtissue fusion into larger tissues

**Fig. 6:**
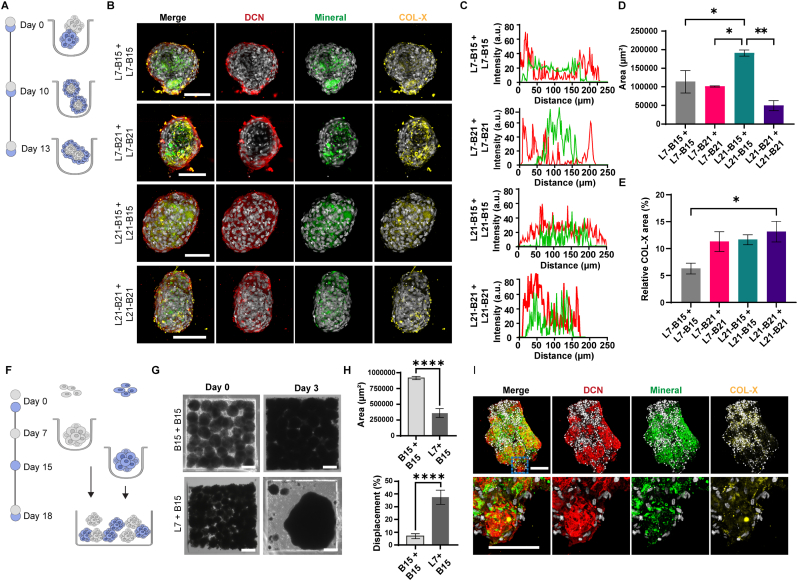
**Fusion of ligament and bone spheroids into large micro- and minitissues. A)** Schematic representation of the timeline of the experiment for creating large microtissues through the fusion of multicellular aggregates within microwell arrays. **B)** Maximum intensity projections of confocal fluorescence microscopy images of large multicellular microtissues. Samples were labeled for decorin (DCN; red), mineral (green), and collagen type X (COL-X; yellow), and counterstained with DAPI to visualize cell nuclei (gray). Scale bars represent 100 μm and apply to all images in the same row. **C)** Fluorescence intensity profiles showing the spatial distribution of DCN (red) and OsteoImage staining (green) across microtissues. Fluorescence intensity is shown in arbitrary units (a.u.). **D)** Bar graphs showing microtissue area after 3 days of culture. Data were analyzed using the Kruskal-Wallis test, followed by Dunn's post hoc test for multiple comparisons. ∗p < 0.05, ∗∗p < 0.01. N = 3. **E)** Bar graphs showing COL-X quantification in the microtissues after 3 days of culture. Data were analyzed using the Kruskal-Wallis test, followed by Dunn's post hoc test for multiple comparisons. ∗p < 0.05. N = 3. **F)** Schematic representation of the experimental timeline for generating minitissues via the fusion of spheroids in defined geometric arrays. **G)** Representative bright-field images of B15 + B15 spheroids (top) and L7 + B15 spheroids (bottom) within the geometric arrays immediately after seeding (left) and after 3 days of culture (right). Scale bars represent 200 μm. **H)** Bar graphs showing projection area (top) and tissue displacement (bottom) of minitissues generated from B15 spheroids or from the co-culture of L7 and B15 spheroids (L7-B15) after 3 days of culture. Normality was assessed by the Shapiro-Wilk test, and group differences were tested using Welch's *t*-test. ∗∗∗∗p < 0.0001. N = 5. **I)** Maximum intensity projections of confocal fluorescence microscopy images of L7 + B15 minitissues after 3 days of culture in the geometric arrays (top) and zoom-in of the blue dotted area (bottom). Samples were stained for DCN (red), OsteoImage to visualize the mineral (green), and COL-X (yellow), and counterstained with DAPI to visualize cell nuclei (gray). Scale bars represent 100 μm and apply to all images in the same row. (For interpretation of the references to color in this figure legend, the reader is referred to the Web version of this article.)

As one of the main aims of tissue engineering and regenerative medicine is to develop scalable approaches for engineering enlarged tissue structures, we explored whether the multicellular aggregates could form larger microtissues once brought into proximity. First, we seeded two multicellular aggregates of the same spheroid pair within each microwell and cultured them for 3 days (Fig. 6A). In all four combinations of spheroids, we observed that the microtissues settled, came into contact with each other, and spontaneously self-assembled to form larger tissue structures (Fig. 6B). Fluorescence microscopy images showed comparable, self-sorted tissue organization across conditions, with the mineralized matrix forming a core surrounded by DCN-expressing cells and COL-X deposits (Fig. 6B and C). When fusing L7-B15 + L7-B15 and L7-B21 + L7-B21, the resulting microtissues formed a ring-like structure around the osteogenic region, similar to their core-shell-structured starting aggregates. By contrast, when fusing L21-B15 + L21-B15 and L21-B21 + L21-B21, the ligament spheroids were more homogeneously distributed in the resulting microtissue. This difference could be related to the amount of ECM produced by ACL spheroids during their pre-incubation culture time (7 days versus 21 days), suggesting that high ECM density might affect cell motility [60]. We also noticed that in three of the four conditions analyzed, with L21-B21 + L21-B21 being the exception, osteogenic spheroids aggregated at the center of the microtissue but did not completely fuse. In contrast, the L21-B21 + L21-B21 assemblies showed a uniform bone-like core. The projection area of the resulting microtissues was smaller in mature L21-B21 + L21-B21 combinations, while we measured a 2-fold area increase in L21-B15 + L21-B15 when compared to the other conditions (Fig. 6D).

Quantification of COL-X in fluorescence microscopy images showed that the area occupied by COL-X deposits was significantly lower in early-stage microtissues (L7-B15 + L7-B15) when compared to the more mature ones (L21-B21 + L21-B21; Fig. 6E). These observations align with our above findings regarding the fusion of heterotypic spheroids, where we observed a similar trend in COL-X deposition in the less mature aggregates.

Next, we aimed to develop larger tissues. To this end, we fabricated an agarose milliowell array, featuring square cavities measuring 1 mm × 1 mm at the base and 500 μm in height, which we refer to as geometric array (Supplementary Fig. 2). Here, we chose L7-B15 as a spheroid pair to test the scalability and adaptability of our approach.

Following culture in microwell arrays, the individual spheroids were pooled and placed into the geometric array (Fig. 6F). As a result, the spheroids fused into apparent continuous tissues. In B15-only cultures, limited tissue compaction and a defined square shape after 3 days of culture was observed in the geometric array, with a final projection area of approximately 0.9 mm^2^ (Fig. 6G and H). In comparison, over the same 3-day period, the co-culture of L7 and B15 spheroids showed dramatic compaction and loss of the original shape, with a significantly smaller projection area of 300,000 μm^2^. Tissue displacement, measured relative to the initial distance between the center and the border of the tissue, was approximately 7% in single-cell-type fused B15 spheroids after 3 days of culture in the geometric array, while it reached approximately 40% in the L7-B15 co-culture system, a significant 5-fold difference (Fig. 6H). These data suggest that the tissue obtained by co-culture of L7 and B15 spheroids contracted and remodeled.

Next, we evaluated the architectural organization of the L7-B15 co-cultured tissue (Fig. 6I). Using immunofluorescence staining of cryosectioned tissue, we observed that the mineral deposits stained with OsteoImage were mainly concentrated in the inner part of the construct concomitantly with COL-X deposits, which formed a core surrounded by DCN-expressing cells. Similar to the fused spheroid pairs above, the tissues self-sorted into a core-shell structure but at a larger scale.

Overall, this platform provides a controlled and reproducible system for studying tissue remodeling in a 3D context and tracking changes that occur during the fusion process. This information could provide insights into the dynamics of enthesis remodeling and offer the possibility to manipulate culture conditions, such as the types of spheroids used, or to test the effects of various compounds or therapeutic agents on tissue fusion and remodeling. Future work could implement automated spheroid dispensing to precisely control spheroid number and minimize well-to-well variability.

### 3.7 Application of acoustic stimulation on fused aggregates

**Fig. 7:**
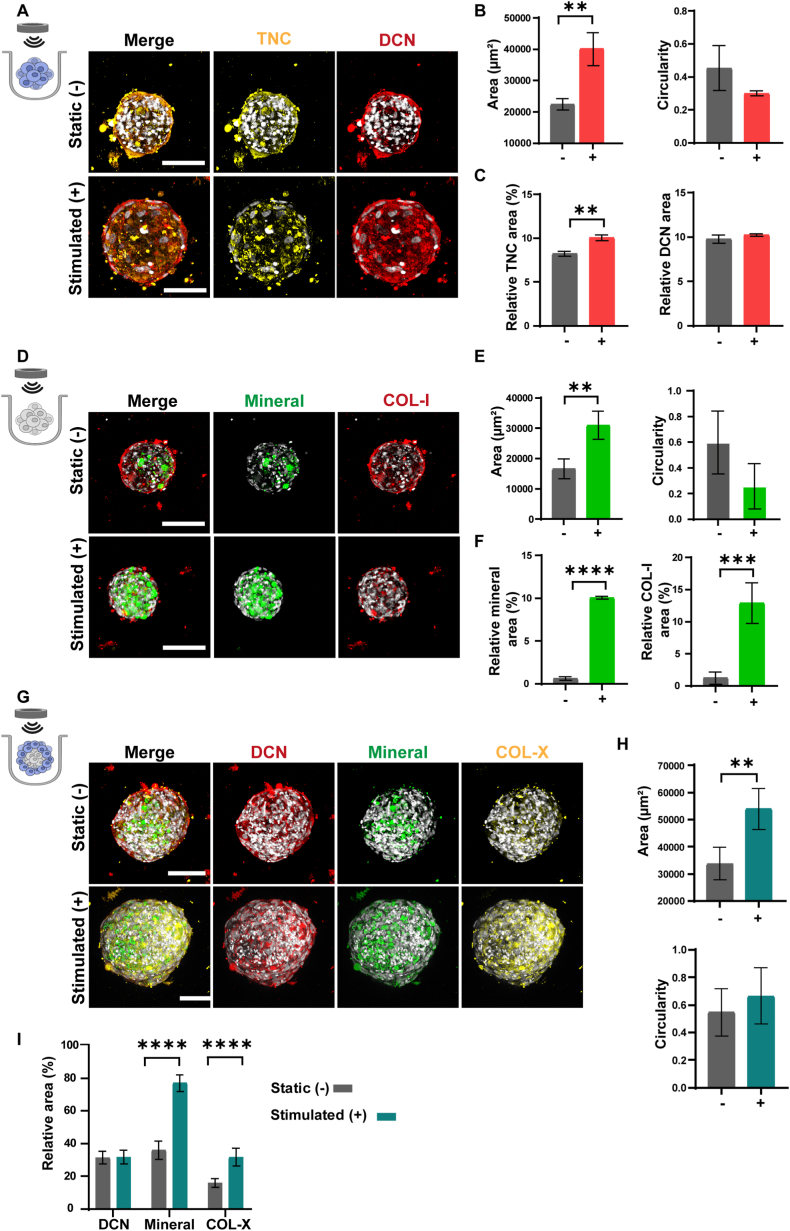
**Effect of acoustic stimulation on L7 spheroids, B15 spheroids and L7-B15 multicellular aggregates. A)** Maximum intensity projections of confocal fluorescence microscopy images of L7 spheroids cultured for 7 days under static (top) and acoustically stimulated (bottom) conditions. Samples were labeled for tenascin C (TNC; yellow) and decorin (DCN; red), and counterstained with DAPI to visualize cell nuclei (gray). Scale bars represent 100 μm and apply to all images in the same row. **B)** Bar graphs showing L7 spheroids' area (left) and circularity (right) after 7 days of culture under static (−) and stimulated (+) conditions. Normality was assessed by the Shapiro-Wilk test, and group differences were tested using Welch's *t*-test. ∗∗p < 0.01. N = 3. **C)** Bar graphs showing TNC and DCN quantification in the L7 spheroids after 7 days of culture under static (−) and stimulated (+) conditions. Normality was assessed by the Shapiro-Wilk test, and group differences were tested using Welch's *t*-test. ∗∗p < 0.01. N = 3. **D)** Maximum intensity projections of confocal fluorescence microscopy images of B15 spheroids cultured for 7 days under static (top) and acoustically stimulated (bottom) conditions. Samples were labeled for mineral (green) and collagen type I (COL-I; red), and counterstained with DAPI to visualize cell nuclei (gray). Scale bars represent 100 μm and apply to all images in the same row. **E)** Bar graphs showing B15 spheroids' area (left) and circularity (right) after 7 days of culture under static (−) and stimulated (+) conditions. Normality was assessed by the Shapiro-Wilk test, and group differences were tested using Welch's *t*-test. ∗∗p < 0.01. N = 3. **F)** Bar graphs showing mineral and COL-I quantification in the B15 spheroids after 7 days of culture under static (−) and stimulated (+) conditions. Normality was assessed by the Shapiro-Wilk test, and group differences were tested using Welch's *t*-test. ∗∗∗∗p < 0.0001. N = 3. **G)** Maximum intensity projections of confocal fluorescence microscopy images of L7-B15 aggregates cultured for 7 days under static (top) and stimulated conditions (bottom). Samples were labeled for DCN (red), mineral (green), and collagen type X (COL-X; yellow), and counterstained with DAPI to visualize cell nuclei (gray). Scale bars represent 100 μm and apply to all images in the same row. **H)** Bar graphs showing spheroid area (top) and circularity (bottom) after 7 days of culture under static (−) and stimulated (+) conditions. Normality was assessed by the Shapiro-Wilk test, and group differences were tested using Welch's *t*-test. ∗∗p < 0.01. N = 3. **I)** Bar graphs showing DCN, mineral, and COL-X quantification in the multicellular aggregates after 7 days of culture under static (−) and stimulated (+) conditions. Normality was assessed by the Shapiro-Wilk test, and group differences were tested using Welch's *t*-test. ∗∗∗∗p < 0.0001. N = 3. (For interpretation of the references to color in this figure legend, the reader is referred to the Web version of this article.)

Considering that cells within the enthesis are subject to dynamic mechanical stimuli, we investigated whether external acoustic stimulation, resulting in mechanical nanovibrations transmitted to the cells, could trigger a response in the fused aggregates (Fig. 7A). To achieve this, we designed a bioreactor set-up consisting of a commercial sound source and a custom-built well insertion [27]. The well insert, fabricated from poly(methyl methacrylate) (PMMA), had an outer diameter of 34.3 mm and a central chamber with a diameter of 15 mm, designed to accommodate the PC microwell arrays (Supplementary Fig. 3).

We first assessed the effect of acoustic stimulation on L7 and B15 spheroids separately (Fig. 7A–F). After culturing in microwells, the L7 and B15 spheroids were transferred to the bioreactor system and subjected to daily acoustic stimulation at 3 kHz for 4 h per day over 7 days. Control spheroids were cultured without stimulation. The spheroids were characterized 12 h after the last acoustic stimulation.

Stimulated L7 spheroids showed a significant 2-fold increase in projection area compared to non-stimulated ones, with no differences in circularity (Fig. 7B). Quantitative analysis revealed a significant increase in tenascin (TNC) expression in acoustically stimulated spheroids compared to non-stimulated ones, while DCN deposition remained unchanged (Fig. 7C). This observation aligns with previous studies showing that TNC is upregulated in fibroblasts following mechanical stimulation and is highly expressed during tissue remodeling [61,62].

For B15 spheroids, external acoustic stimulation produced similar effects, nearly doubling the projection area compared to non-stimulated cultures (Fig. 7E). Circularity remained consistent between conditions, suggesting that acoustic stimulation did not alter the formation of spherical aggregates. However, the application of sound waves significantly increased mineral deposition, with 16 times more positive OsteoImage staining, and formed a denser COL-I network compared to controls, suggesting enhanced mineral formation (Fig. 7F). Because the B15 spheroids were cultured alone, COL-I provides an unambiguous osteogenic matrix readout (unlike in fused constructs), and acoustic/ultrasound stimulation has been reported to increase COL-I deposition in osteogenic environments [[63], [64], [65]]. In line with our data, acoustic stimulation was previously shown to promote osteogenic differentiation of human bone marrow stromal cells (hBMSCs) cultured in 3D scaffolds [66], enhance calcium deposition and enrich the synthesis of matrix proteins supporting mineralization in MC3T3-E1 osteoblasts [67]. In conclusion, our data suggest that acoustic stimulation during the culture of single-cell-type spheroids led to increased size and enhanced matrix deposition.

Next, we co-cultured L7 and B15 spheroids in the same microwell arrays and, after 24 h, exposed them to external acoustic stimulation (3 kHz, 4 h per day) for 7 days (Fig. 7G). After 7 days of culture, the projection area of the multicellular aggregates was significantly increased, tripling in size compared to non-stimulated controls, while circularity values remained similar across conditions (Fig. 7H). We hypothesized that this change might be due to enhanced ECM deposition and/or increased cell proliferation, which helped the aggregates maintain their original spherical shape. In fact, previous studies demonstrated that low-intensity ultrasound stimulation increased matrix production in fibroblasts [68], and cell proliferation in skin fibroblasts [69]. Moreover, following quantification of immunostaining for the ECM components, we noted a significantly increased deposition of mineral-containing ECM, as well as a significantly enhanced area of COL-X network in acoustically stimulated cultures compared to non-stimulated ones (Fig. 7I).

We also noted a different matrix distribution in L7 and B15 spheroids in the multicellular aggregates (Fig. 7G). While a core-shell organization was prevalent in statically cultured samples, a homogeneous mix of mineral and DCN-enriched ECM was found in the acoustically stimulated aggregates. This shift in organization might result from changes in the tissue-cohesive properties due to the stimulation. The increased accumulation of ECM molecules could affect the fusion kinetics and spheroid engulfing, leading to a more disorganized mechanism in stimulated samples [70]. Further studies on the correlation of the enveloping behavior of tissue spheroids combined with measurement of their surface tension and viscosity may elucidate the biophysical basis for our observations.

Further studies are necessary to unravel the molecular mechanisms involved in the response of multicellular aggregates to external acoustic stimulations. Some reports have shown that the activation of L-type voltage-gated Ca^2+^ ion channels in hBMSCs regulates calcium influx into the cells, playing a pivotal role in cell attachment, proliferation and osteogenic differentiation [66]. Understanding the biological programs activated by acoustic stimulation might also facilitate the development of new technologies for the therapeutic treatment of enthesis-related diseases. In particular, identifying which cell populations and pathways respond to acoustic stimulation in a spatially organized interface model could inform how such stimuli can be tailored to promote beneficial remodeling at the tendon-bone junction. Indeed, several clinical studies have already tested low-intensity pulsed ultrasound for the treatment of osteoarthritis [71], or bone fractures [72], but have not focused on the enthesis.

## 4 Conclusion and outlook

In conclusion, we present a versatile spheroid-fusion strategy for fabricating 3D, scaffold-free aggregates that replicate key structural and molecular features of the enthesis. By combining ACL-derived and hMSC-derived spheroids, we generate spatially organized ligament-to-bone-like constructs. They are characterized by (i) a graded transition from ligament-like ECM characterized by TNC expression to a mineralizing fibrocartilage-like region characterized by COL-X expression, and (ii) the formation of a mineralized zone supported by calcium-rich deposition and RUNX2 upregulation at both gene and protein level. These hallmark features demonstrate the capacity of our model to reproduce essential aspects of the native enthesis interface. The polarized microtissues hold potential for both basic and translational applications. The reproducibility and scalability of the platform make it suitable for the integration of quantitative mechanical assays to determine how interface properties evolve with maturation and mechanical stimulation. In addition, the system provides a controlled framework to investigate enthesis-related pathophysiologies, such as inflammation-driven fibrocartilage degradation and aberrant mineralization associated with tendinopathy and enthesopathy. The modular and scaffold-free nature of the multicellular microtissues makes them promising candidates for injectable or bioprintable building blocks in future enthesis repair strategies. Moreover, the heterotypic spheroid-fusion protocol is compatible with microwell array systems, enabling high-throughput screening of bioactive cues that promote zone-specific regeneration or modulate inflammatory responses. Together, these capabilities position the presented approach as a valuable addition for engineering and studying complex musculoskeletal interfaces.

## CRediT authorship contribution statement

**Francesca Giacomini:** Conceptualization, Formal analysis, Investigation, Methodology, Validation, Visualization, Writing – original draft, Writing – review & editing. **Shivesh Anand:** Investigation, Methodology, Validation, Writing – original draft, Writing – review & editing. **Steven Vermeulen:** Investigation, Writing – review & editing. **David Barata:** Investigation, Writing – review & editing. **Zeinab Niloofar Tahmasebi Birgani:** Supervision, Writing – review & editing. **Pieter J. Emans:** Investigation, Writing – review & editing. **Carmen López-Iglesias:** Investigation, Writing – review & editing. **Lorenzo Moroni:** Supervision, Writing – review & editing. **Carlos Mota:** Supervision, Writing – review & editing. **Stefan Giselbrecht:** Conceptualization, Funding acquisition, Methodology, Supervision, Writing – review & editing. **Pamela Habibović:** Conceptualization, Funding acquisition, Methodology, Supervision, Validation, Writing – review & editing. **Roman Truckenmüller:** Conceptualization, Funding acquisition, Methodology, Supervision, Validation, Writing – review & editing.

## Declaration of competing interest

The authors declare the following financial interests/personal relationships which may be considered as potential competing interests: Stefan Giselbrecht reports a relationship with 300MICRONS GmbH that includes: board membership and equity or stocks. Roman Truckenmueller reports a relationship with 300MICRONS GmbH that includes: board membership and equity or stocks. Shivesh Anand has patent Biomimetic bioreactor for biomimetic stimulation, method for forming tissue in such a biomimetic bioreactor and sample holder suitable for use in such a reactor pending to Maastricht University and Maastricht University Medical Center+. Carlos Mota has patent Biomimetic bioreactor for biomimetic stimulation, method for forming tissue in such a biomimetic bioreactor and sample holder suitable for use in such a reactor pending to Maastricht University and Maastricht University Medical Center+. Roman Truckenmueller and Stefan Giselbrecht are founders, shareholders, and managing directors of 300MICRONS GmbH, a company active in the field of microengineered 3D cell culture solutions. If there are other authors, they declare that they have no known competing financial interests or personal relationships that could have appeared to influence the work reported in this paper.

## Data Availability

Data will be made available on request.
