# Supplementary material for: Multicellular microtissues from fused ligament- and bone-cell spheroids relevant to enthesis repair
Source: Mater Today Bio. 2026 Apr 15;38:103129. doi: 10.1016/j.mtbio.2026.103129 (PMC13141807; doi:10.1016/j.mtbio.2026.103129)
Supplement: Multimedia component 1 [file mmc1.docx]

Supplementary data

**Multicellular microtissues from fused ligament- and bone-cell spheroids relevant to enthesis repair**

Francesca Giacomini, Shivesh Anand, Steven Vermeulen, David Barata, Zeinab Niloofar Tahmasebi Birgani, Pieter J. Emans, Carmen López-Iglesias, Lorenzo Moroni, Carlos Mota, Stefan Giselbrecht, Pamela Habibović, Roman Truckenmüller


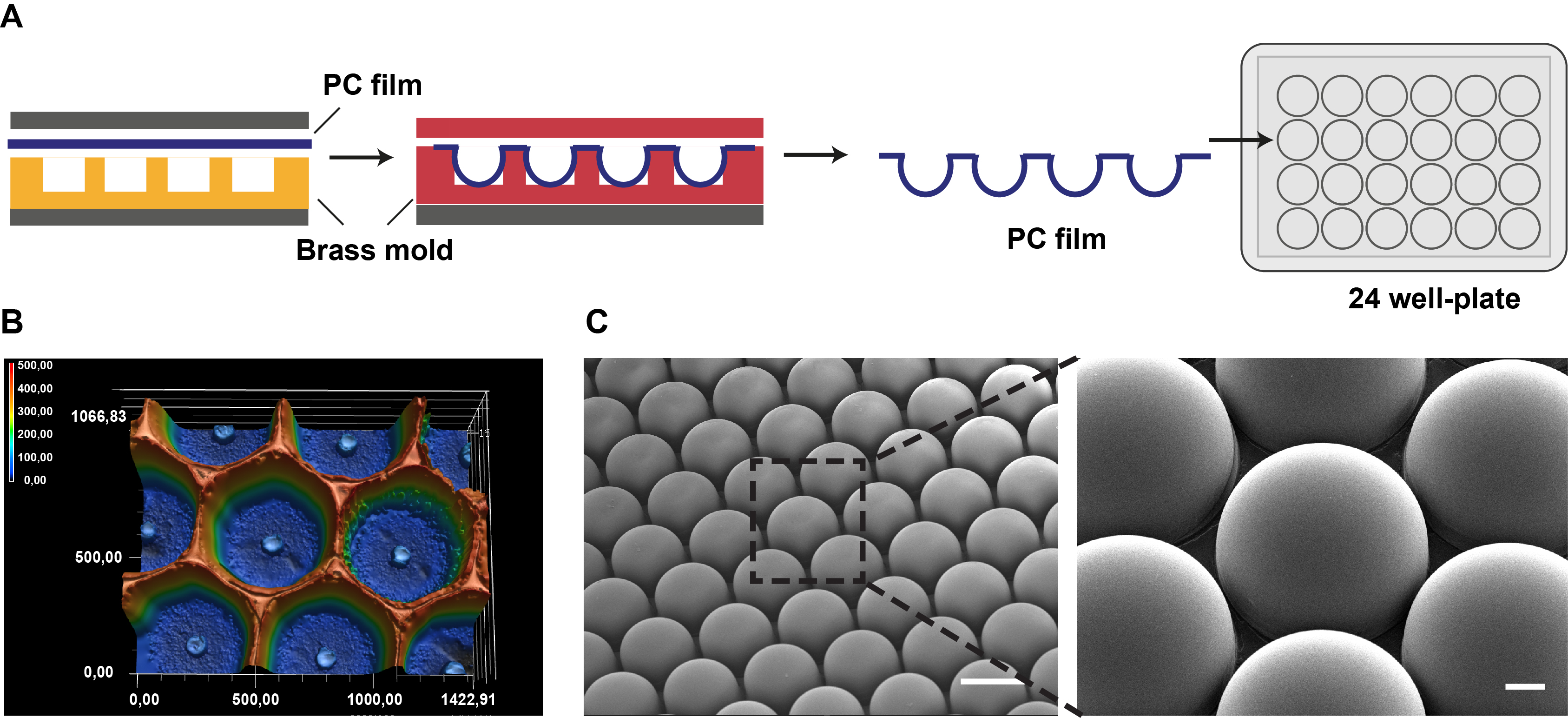


**Supplementary Fig. 1. Fabrication and characterization of microwell arrays.** **A)** Schematic representation of the fabrication process for the polycarbonate (PC) microwell array using gas-assisted microthermoforming. **B)** Confocal laser scanning profilometry analysis showing the 3D reconstruction of a part of the microwell array. All scales are in µm. **C)** Scanning electron microscopy images of the microwell array. Scale bars represent 500 µm (left image) and 100 µm (right image).


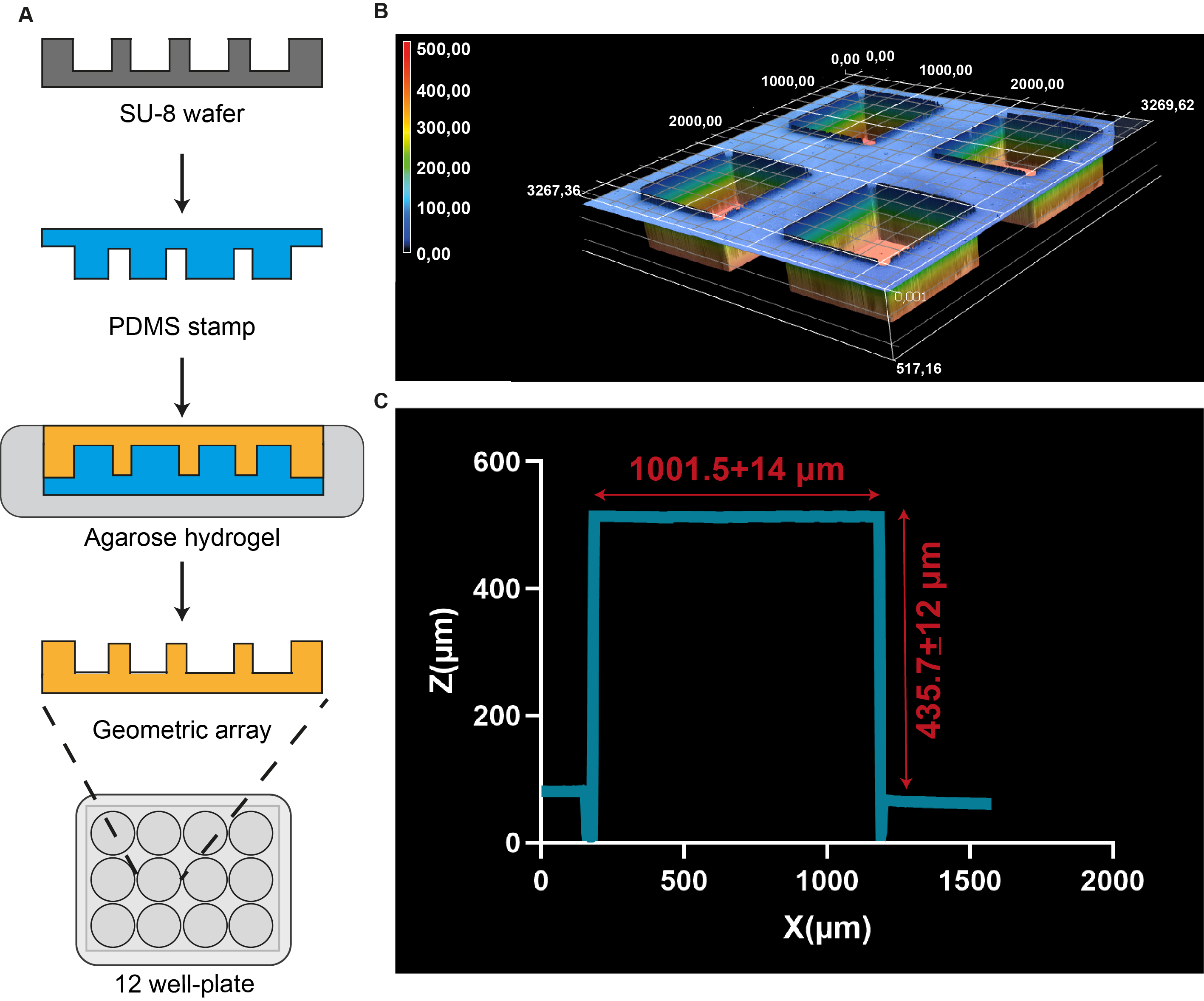


**Supplementary Fig. 2. Fabrication and characterization of geometric arrays.** **A)** Schematic representation of the fabrication of the geometric array via replica molding in agarose hydrogel used for the fabrication of enlarged tissues. **B)** Confocal laser scanning profilometry analysis showing the 3D reconstruction of one part of the SU-8-silicon wafer-based lithographic master for fabricating the geometric array. All scales are in µm. **C)** Confocal laser scanning profilometry analysis showing the (2D) profile of one part of the lithographic master.


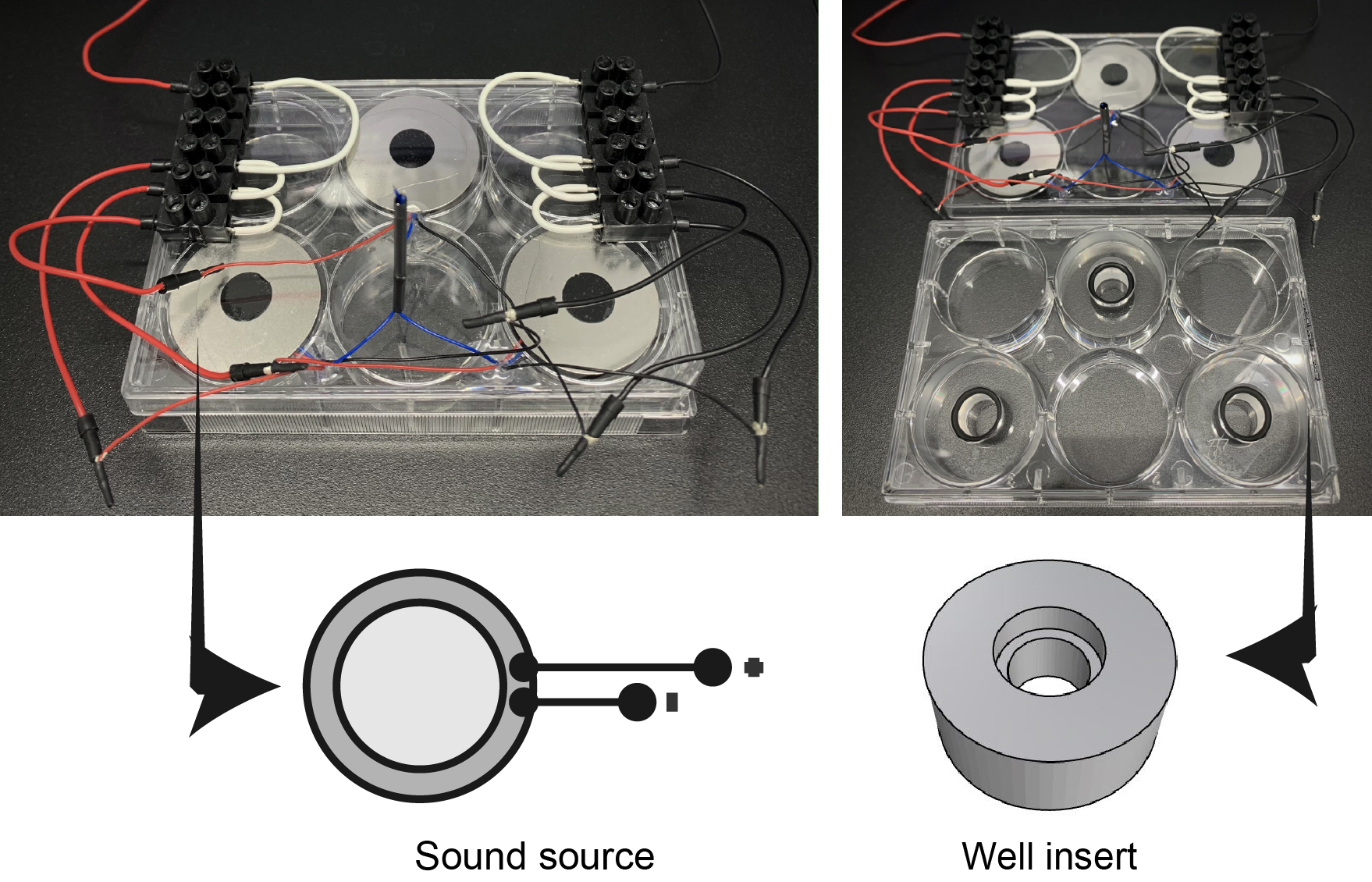


**Supplementary Fig. 3. Acoustic stimulation setup.** Bioreactor set-up used for acoustic stimulation. The bioreactor consists of a poly(methyl methacrylate) (PMMA) housing with an internal diameter of 15 mm, where the polycarbonate (PC) microwell array was placed for culturing multicellular aggregates. The PMMA housing was then placed in a 6 well-plate.


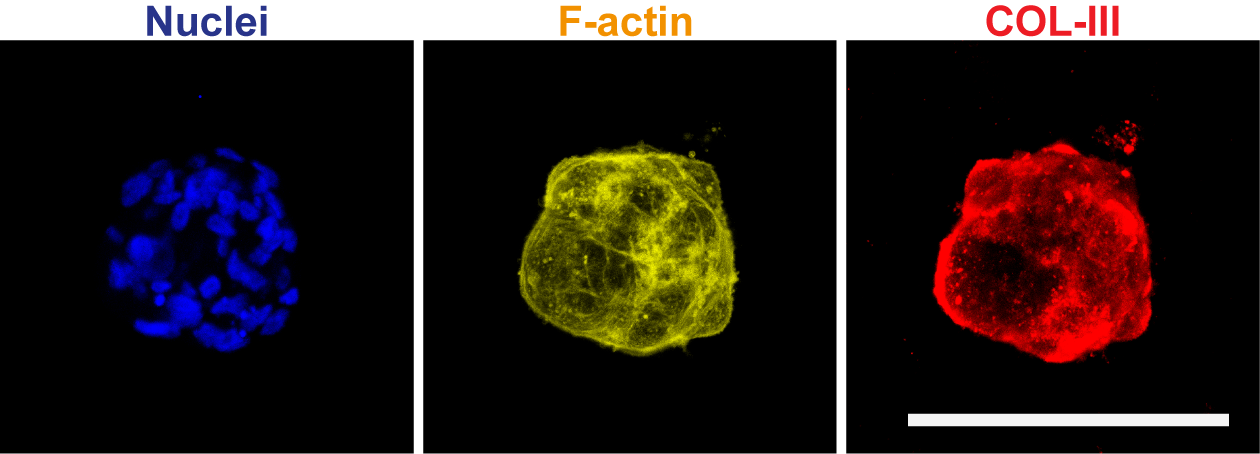


**Supplementary Fig. 4. Nuclear and actin staining of anterior cruciate ligament (ACL) spheroids.** Representative fluorescence images of an ACL spheroid on day 21 of culture stained for nuclei (DAPI; blue), F-actin (yellow), and collagen type III (COL-III; red). These images provide morphological context for the ACL spheroids corresponding to Fig. 2F. Scale bar represents 100 µm and applies to all images.


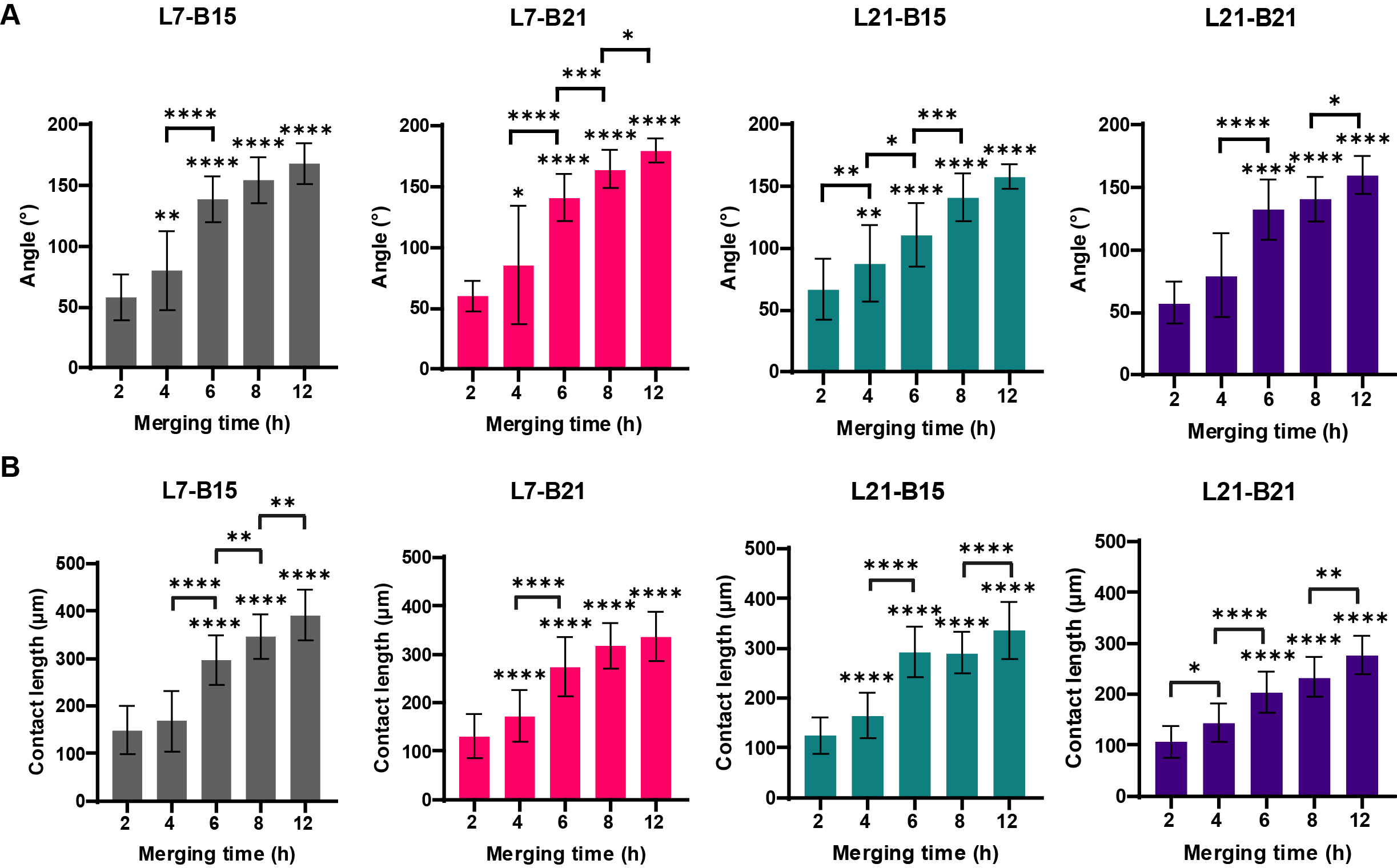


**Supplementary Fig. 5**. **Fusion kinetics of anterior cruciate ligament and bone spheroids**. **A)** Bar graphs showing the tangent angles between L7-B15 (gray), L7-B21 (pink), L21-B15 (green) and L21-B21 (purple) spheroid pairs over time. Data were analyzed using the Kruskal-Wallis test followed by Dunn’s post hoc test for multiple comparisons. Asterisks directly above the bars indicate statistical significance relative to 2 h time point. * p < 0.05, ** p < 0.01, *** p < 0.001, **** p < 0.0001. N = 20. **B)** Bar graphs showing the contact length between L7-B15 (gray), L7-B21 (pink), L21-B15 (green) and L21-B21 (purple) spheroid pairs over time. Data were analyzed using the Kruskal-Wallis test followed by Dunn’s post hoc test for multiple comparisons. Asterisks directly above the bars indicate statistical significance relative to 2 h time point. * p < 0.05, ** p < 0.01, *** p < 0.001, **** p < 0.0001. N = 20.


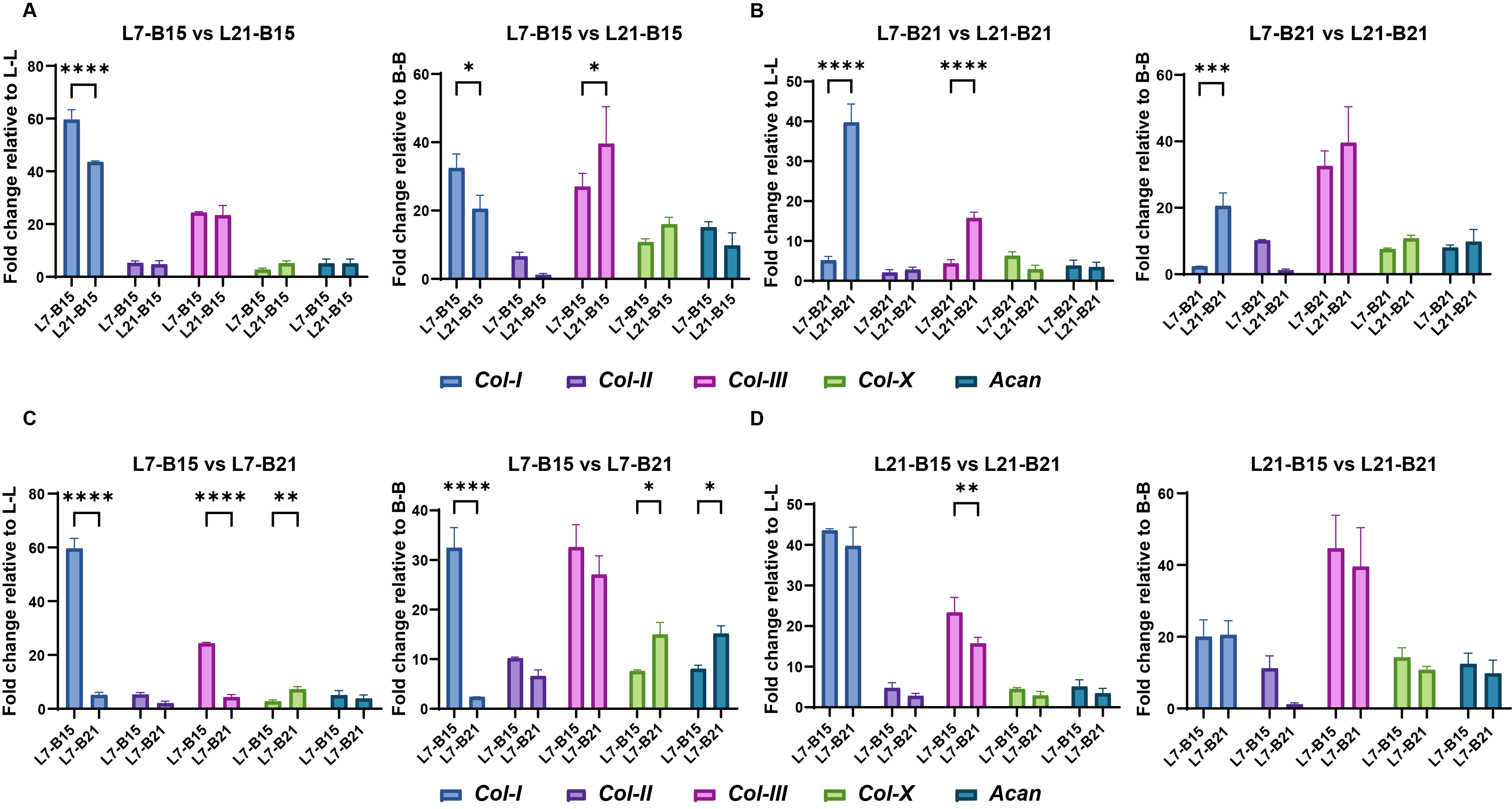


**Supplementary Fig. 6. Chronological expression of enthesis-related markers.** **A** and **B)** Bar graphs showing the comparison of the expression of the enthesis-related genes collagen type I (*Col-I*; blue), type II (*Col-II*; purple), type III (*Col-III*; pink), type X (*Col-X*; green), and aggrecan (*Acan*; cyan) in (A) L7-B15 and L21-B15 aggregates and (B) L7-B21 and L21-B21 aggregates to assess the effect of anterior cruciate ligament (ACL) spheroid maturation. Values are given as fold changes relative to homotypic ACL (L-L, left) and bone (B-B, right) spheroid pairs of the same maturity stage. Data were analyzed using the Kruskal-Wallis test followed by Dunn’s post hoc test for multiple comparisons. * p < 0.05, *** p < 0.001, **** p < 0.0001. N = 3. **C** and **D)** Bar graphs showing the comparison of the expression of the enthesis-related genes *Col-I* (blue), *Col-II* (purple), *Col-III* (pink), *Col-X* (green), and *Acan* (cyan) in (C) L7-B15 and L7-B21 aggregates and (D) L21-B15 and L21-B21 aggregates to assess the effect of bone spheroid maturation. Values are given as fold changes relative to homotypic ACL (L-L, left) and bone (B-B, right) spheroid pairs of the same maturity stage. Data were analyzed using the Kruskal-Wallis test followed by Dunn’s post hoc test for multiple comparisons. * p < 0.05, ** p < 0.01, *** p < 0.001, **** p < 0.0001. N = 3.


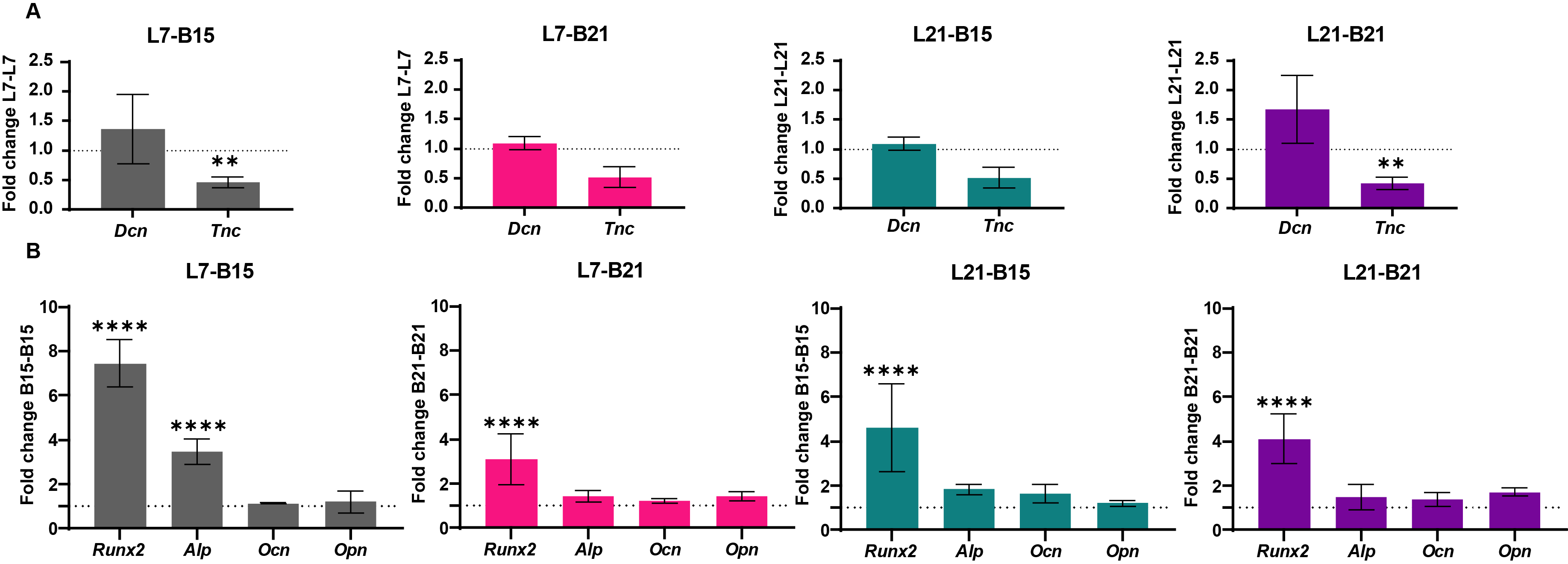


**Supplementary Fig. 7. Expression of ligament and osteogenic markers in fused spheroid pairs.** **A)** Bar graphs showing the expression of the ligament-related genes decorin (*Dcn*) and tenascin C (*Tnc*) in L7-B15 (gray), L7-B21 (pink), L21-B15 (green), and L21-B21 (purple) aggregates. Values are given as fold change relative to fused anterior cruciate ligament spheroids (L-L) at the same maturity stage, represented as a dotted line. Normality was assessed by the Shapiro-Wilk test, and group differences were tested using Welch’s t-test. ** p < 0.01, **** p < 0.0001. N = 3. **B)** Bar graphs showing the expression of the osteogenic-related genes Runt-related transcription factor 2 (*Runx2*), alkaline phosphatase (*Alp*), osteocalcin (*Ocn*), and osteopontin (*Opn*) in L7-B15 (gray), L7-B21 (pink), L21-B15 (green), and L21-B21 (purple) aggregates. Values are given as fold change relative to fused bone spheroids (B-B) at the same maturity stage, represented as a dotted line. Data were analyzed using the Kruskal-Wallis test, followed by Dunn’s post hoc test for multiple comparisons. ** p < 0.01, **** p < 0.0001. N = 3.


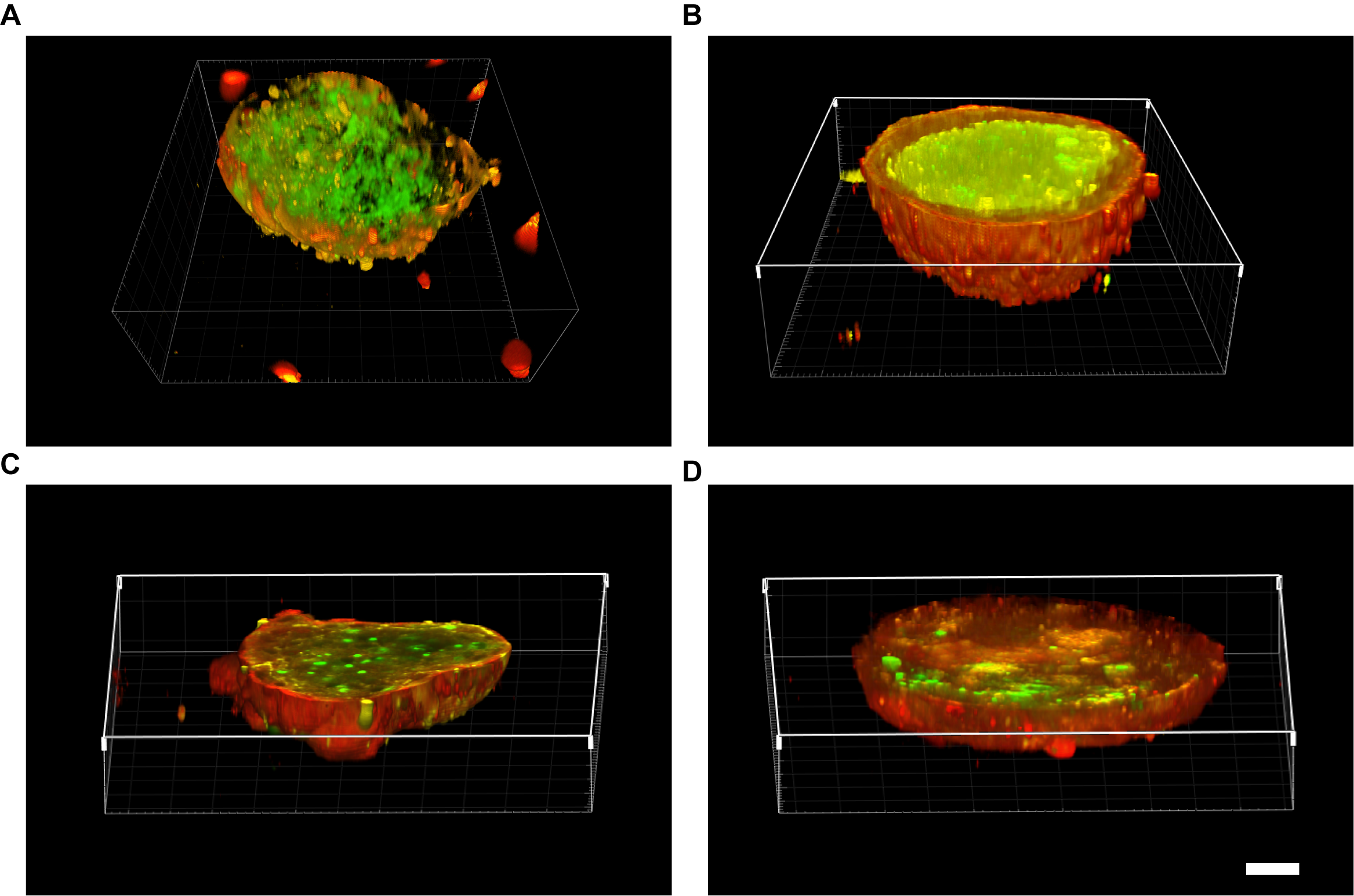


**Supplementary Fig. 8. Central optical sections in fused microtissues.** Representative confocal z-stack slices (mid-stack) of fused microtissues after 10 days of co-culture stained for decorin (DCN; ligament-like matrix; red), mineral (OsteoImage; green), and collagen type X (COL-X; fibrocartilage-like matrix; yellow) in **A)** L7-B15, **B)** L7-B21, **C)** L21-B15, and **D)** L21-B21 aggregates. Scale bar represents 50 µm and applies to all images.


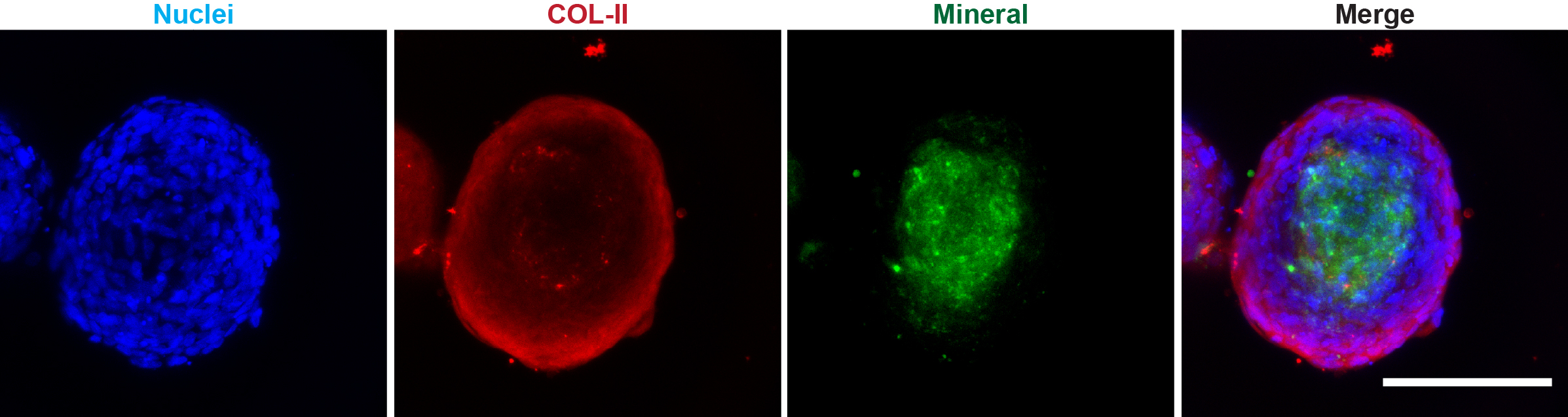


**Supplementary Fig. 9. Collagen type II (COL-II) staining in fused microtissues (L7-B21).** Representative confocal fluorescence images (maximum-intensity projections) of an L7-B21 fused microtissue after 10 days of co-culture stained for cell nuclei (blue), COL-II (red), and mineral (OsteoImage; green), with the merged image displayed on the right. Scale bar represents 100 µm and applies to all images.


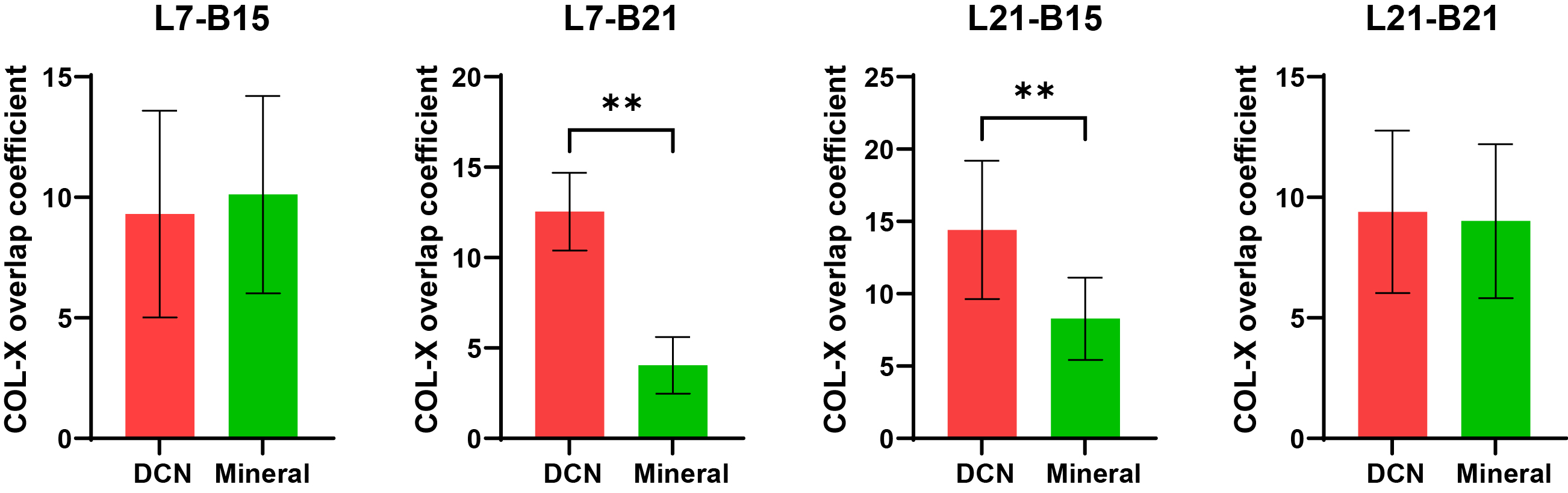


**Supplementary Fig. 10. Collagen type X (COL-X) overlap coefficient in the multicellular aggregates.** Bar graphs showing the overlap coefficient between COL-X- and decorin (DCN)- or mineral-containing matrix measured as the percentage of co-localized pixels between the respective signals in the multicellular aggregates. Normality was assessed by the Shapiro-Wilk test, and group differences were tested using Welch’s t-test. ** p < 0.01. N = 10.

**Supplementary Table 1.** Primary antibodies and their dilutions.

| **Primary antibody** | **Dilution** | **Supplier** | **Catalogue number** |
| --- | --- | --- | --- |
| Rabbit anti Collagen-III | 1:100 | Abcam | ab7778 |
| Rabbit anti Decorin | 1:100 | Abcam | ab175404 |
| Mouse anti Tenascin C | 1:100 | Thermo Fisher Scientific | MA5-16086 |
| Mouse anti Runx2 | 1:100 | Abcam | ab76956 |
| Rabbit anti Collagen-I | 1:100 | Abcam | ab34710 |
| Rabbit anti Osteocalcin | 1:50 | Abcam | ab93876 |
| Mouse anti Collagen-X | 1:50 | Abcam | ab49945 |

**Supplementary Table 2.** Secondary antibodies and their dilutions.

| **Primary antibody** | **Secondary antibody** | **Dilution** | **Supplier** |
| --- | --- | --- | --- |
| Rabbit anti Collagen-III | Alexa Fluor-647  goat anti-rabbit | 1:500 | Thermo Fisher Scientific |
| Rabbit anti Decorin | Alexa Fluor-647  goat anti-rabbit | 1:500 | Thermo Fisher Scientific |
| Mouse anti Tenascin C | Alexa Fluor-488  goat anti-mouse | 1:500 | Thermo Fisher Scientific |
| Mouse anti Runx2 | Alexa Fluor-568  goat anti-mouse | 1:500 | Thermo Fisher Scientific |
| Rabbit anti Collagen-I | Alexa Fluor-647  goat anti-rabbit | 1:500 | Thermo Fisher Scientific |
| Rabbit anti Osteocalcin | Alexa Fluor-488  goat anti-rabbit | 1:500 | Thermo Fisher Scientific |
| Mouse anti Collagen-X | Alexa Fluor-568  goat anti-mouse | 1:500 | Thermo Fisher Scientific |

**Supplementary Table 3.** Primer sequences used for RT-qPCR.

| **Gene** | **Forward primer** | **Reverse primer** |
| --- | --- | --- |
| *Col-III* | TACTTCTCGCTCTGCTTCATCC | GAACGGATCCTGAGTCACAGAC |
| *Dcn* | TGGGCTGGCAGAGCATAAGT | CCAGGTGGGCAGAAGTCACT |
| *Tnc* | ATGTCCTCCTGACAGCCGAGAA | AGTCACGGTGAGGTTTTCCAGC |
| *Col-I* | AGCGGACGCTAACCCCCTC | CAGACGGGACAGCACTCGCC |
| *Runx2* | CCCAGTATGAGAGTAGGTGTCC | GGGTAAGACTGGTCATAGGACC |
| *Alp* | ATCTTTGGTCTGGCCCCCATG | AGTCCACCATGGAGACATTCTCTC |
| *Ocn* | TCACACTCCTCGCCCTATTG | GAAGAGGAAAGAAGGGTGCC |
| *Opn* | TCACCAGTCTGATGAGTCTCACCATTC | TAGCATCAGGGTACTGGATGTCAGGT |
| *Col-II* | AACCAGATTGAGAGCATCCG | ACCTTCATGGCGTCCAAG |
| *Col-X* | CCCTCTTGTTAGTGCCAACC | AGATTCCAGTCCTTGGGTCA |
| *Acan* | AGTAGAGGACATCAGCGGGCTT | CCGCTGATGTCCTCTACTCCAG |
| *Gapdh* | GGAGCGAGATCCCTCCAAAAT | GGCTGTTGTCATACTTCTCATGG |
| *18S rRNA* | GTAACCCGTTGAACCCCATT | CCATCCAATCGGTAGTAGCG |
